# The vertebrate ancestral repertoire of visual opsins, transducin alpha subunits and oxytocin/vasopressin receptors was established by duplication of their shared genomic region in the two rounds of early vertebrate genome duplications

**DOI:** 10.1186/1471-2148-13-238

**Published:** 2013-11-02

**Authors:** David Lagman, Daniel Ocampo Daza, Jenny Widmark, Xesús M Abalo, Görel Sundström, Dan Larhammar

**Affiliations:** 1 Department of Neuroscience, Science for Life Laboratory, Uppsala University, Box 593, SE-75124 Uppsala, Sweden; 2 Present address: Department of Medical Biochemistry and Microbiology, Science for Life Laboratory, Uppsala University, Box 582, SE-75123 Uppsala, Sweden

**Keywords:** Visual opsins, Whole genome duplications, Chromosome rearrangements, Opsin evolution, Oxytocin receptors, Vasopressin receptors, G-protein alpha transducing subunits, Voltage-gated calcium channels

## Abstract

**Background:**

Vertebrate color vision is dependent on four major color opsin subtypes: RH2 (green opsin), SWS1 (ultraviolet opsin), SWS2 (blue opsin), and LWS (red opsin). Together with the dim-light receptor rhodopsin (RH1), these form the family of vertebrate visual opsins. Vertebrate genomes contain many multi-membered gene families that can largely be explained by the two rounds of whole genome duplication (WGD) in the vertebrate ancestor (2R) followed by a third round in the teleost ancestor (3R). Related chromosome regions resulting from WGD or block duplications are said to form a paralogon. We describe here a paralogon containing the genes for visual opsins, the G-protein alpha subunit families for transducin (GNAT) and adenylyl cyclase inhibition (GNAI), the oxytocin and vasopressin receptors (OT/VP-R), and the L-type voltage-gated calcium channels (CACNA1-L).

**Results:**

Sequence-based phylogenies and analyses of conserved synteny show that the above-mentioned gene families, and many neighboring gene families, expanded in the early vertebrate WGDs. This allows us to deduce the following evolutionary scenario: The vertebrate ancestor had a chromosome containing the genes for two visual opsins, one GNAT, one GNAI, two OT/VP-Rs and one CACNA1-L gene. This chromosome was quadrupled in 2R. Subsequent gene losses resulted in a set of five visual opsin genes, three GNAT and GNAI genes, six OT/VP-R genes and four CACNA1-L genes. These regions were duplicated again in 3R resulting in additional teleost genes for some of the families. Major chromosomal rearrangements have taken place in the teleost genomes. By comparison with the corresponding chromosomal regions in the spotted gar, which diverged prior to 3R, we could time these rearrangements to post-3R.

**Conclusions:**

We present an extensive analysis of the paralogon housing the visual opsin, GNAT and GNAI, OT/VP-R, and CACNA1-L gene families. The combined data imply that the early vertebrate WGD events contributed to the evolution of vision and the other neuronal and neuroendocrine functions exerted by the proteins encoded by these gene families. In pouched lamprey all five visual opsin genes have previously been identified, suggesting that lampreys diverged from the jawed vertebrates after 2R.

## Background

Color vision is the ability to distinguish light of different wavelengths. This property has arisen independently in vertebrates and invertebrates through duplications of the genes encoding ciliary opsins and rhabdomeric opsins, respectively [1]. In the vertebrates, three types of cones displaying distinct wavelength properties were initially described by Ragnar Granit who received the Nobel Prize for these achievements in 1967. Later studies, including molecular cloning of the visual opsins in various vertebrates, have shown that four major color opsin subtypes existed in early vertebrates [2-4]. Thus, together with the dim-light (scotopic) receptor rhodopsin expressed in rods, the family of visual opsins encompassed five members in early vertebrates: *RH1* (rhodopsin), *RH2* (green opsin), *SWS1* (ultraviolet opsin), *SWS2* (blue opsin), and *LWS* (red opsin).

Subsequently, the gene repertoire has changed by gains and losses of opsin genes in the different vertebrate lineages. For instance, the ancestor of placental mammals lost *RH2* and *SWS2* and extant mammals use *SWS1* for vision in the blue part of the spectrum. Primates have a duplicate of *LWS* that has mutated to have its absorption maximum in green, namely *OPN1MW*. The platypus has retained *SWS2* but lost *SWS1*. Other losses have occurred in frogs (*RH2*), in the coelacanth *Latimeria chalumnae*, and in cartilaginous fishes. In teleost fishes, on the other hand, many additional duplicates have arisen [5].

Phylogenetic analyses of the five visual opsin sequences in chicken showed that the rhodopsin sequence *RH1* was most closely related to the green opsin *RH2*, suggesting that this gene duplication was the most recent and that the four color opsins existed before rhodopsin [6]. Thus, it appeared that color vision was ancestral to the dim-light vision and the appearance of rhodopsin and rod photoreceptors facilitated the adaptation to nocturnal environments. As a rhodopsin sequence had already been described in a lamprey, this implied that all five opsins existed before the divergence of lampreys and gnathostomes, i.e., jawed vertebrates. Subsequently, also the four color opsins have been described in pouched lamprey (*Geotria australis*) [7].

Although the visual opsin gene duplications must have taken place before the lamprey-gnathostome divergence, it has remained unclear how they happened. We and others have previously reported that numerous gene families expanded in the two genome doublings, i.e. tetraploidizations, that took place before the radiation of gnathostomes [8-11] usually called 2R for two rounds of genome doubling. The two tetraploidizations resulted in a large number of quartets of related chromosome regions, and each such quartet is called a paralogon. Subsequently, a third tetraploidization, 3R, took place in the lineage leading to teleost fishes [12]. By investigating gene families sharing chromosome regions, we have been able to deduce the evolution of multiple neuronal and endocrine gene families as well as their neighbors, namely the opioid peptides [13] and receptors [14], neuropeptide Y-family peptides [15] and receptors [16,17], voltage-gated sodium channels and their neighboring TGF-β receptors [18], the IGFBP family [19], the paralemmin family [20] and, more recently, the three transducin subunit gene families activated by visual opsins [21]. All of these families received additional members in 2R and all but one expanded further in 3R. Chromosomal positions thereby constitute a useful additional type of information for analyses of gene families, especially families that display different evolutionary rates among members or over time, both of which seem to afflict the visual opsins.

We have previously performed sequence-based phylogenetic analyses of several gene families in the phototransduction cascade, and also investigated their chromosomal positions in the human genome [21-23]. These analyses suggested that most of the phototransduction gene families expanded in the basal vertebrate tetraploidizations, including the visual opsin family. In our first study, we proposed that *RH1*, *SWS1* and *LWS* arose as a result of duplications of a large chromosome block [23]. Because teleost fishes and birds have the *LWS* and *SWS2* genes in close proximity on the same chromosome [24-26], we suggested a scenario where two adjacent visual opsin genes were quadrupled by chromosome duplications [22]. However, the paralogon harboring the opsin genes seemed to have undergone major rearrangements and only a few adjacent gene families were identified, thereby making our conclusions uncertain. Interestingly, one of the neighboring gene families was the transducin alpha subunit family (GNAT), involved in the phototransduction cascade, as well as its adjacent relative G protein alpha inhibiting subunit (GNAI) gene [21-23]. The GNAT family includes three genes located on three of the visual opsin chromosomes in several vertebrates, each flanked by a more distantly related *GNAI* gene [21,27,28].

In parallel, independent analyses in our laboratory of the oxytocin/vasopressin receptor (OT/VP-R) genes and the L-type voltage-gated calcium channel alpha subunit (CACNA1-L) genes converged to reveal large chromosomal regions that share evolutionary history with the visual opsin, *GNAT* and *GNAI* genes. The pituitary peptide hormones oxytocin and vasopressin have previously been reported to have five to six ancestral vertebrate receptors (OT/VP-R) based on phylogenetic analyses [29,30]: one oxytocin receptor, OTR, encoded by *OXTR* genes, and four to five vasopressin receptors, including V1A (*AVPR1A*), V1B (*AVPR1B*) as well as several types of V2 receptors. We report here that the OT/VP-R family genes are located in the proposed visual opsin paralogon, thus resolving the issue of the evolutionary relationships between the ancestral members. Finally, the L-type voltage-gated calcium channel alpha subunits (CACNA1-L) form a family with four members whose genes are located on the visual opsin chromosomes.

We have used these gene families as starting points for extensive analyses of conserved synteny in species representing several vertebrate classes. We report here that these five main gene families and 34 neighboring gene families comprise large paralogous chromosomal regions with extensive similarities to one another that can most parsimoniously be explained by quadruplication of a large ancestral chromosome region. These results define the time points for expansion of the visual opsin family as well as the transducin alpha family, the oxytocin/vasopressin receptors, and the L-type voltage-gated calcium channels. Furthermore, our results have implications for the divergence time point of lampreys and jawed vertebrates relative to the two basal vertebrate tetraploidizations.

## Results

We used amino acid sequences identified in genome databases to produce alignments and phylogenetic trees for the visual opsins, the oxytocin and vasopressin receptors (OT/VP-R), the G-protein alpha transducing (GNAT) and inhibiting (GNAI) subunits, as well as the L-type voltage-gated calcium channel alpha subunits (CACNA1-L). Detailed information on these gene families, including database identifiers, location data, genome assembly information and annotation notes for all identified sequences, is provided in Additional file 1. Topologies of the visual opsin and OT/VP-R gene family trees are presented in this section and we have recently published our phylogenetic analyses of the GNAT and GNAI gene families [21]. The phylogenetic analyses of the CACNA1-L family, which have not been published previously, are included in Additional file 2: Figures S7 and S8. These gene families have members located in overlapping regions of the human genome putatively considered as one paralogon and are henceforth referred to as the “main” gene families of this study.

We have also made phylogenetic trees for 34 neighboring gene families identified in the chromosomal regions of the main gene families. The positional data from the main gene families and the neighboring gene families have been compared between the genomes of human, chicken, zebrafish, three-spined stickleback and spotted gar. This combination of phylogenetic and synteny data is the basis for the description of our results below.

### Phylogenetic analyses of vertebrate visual opsin genes

**Figure 1:**
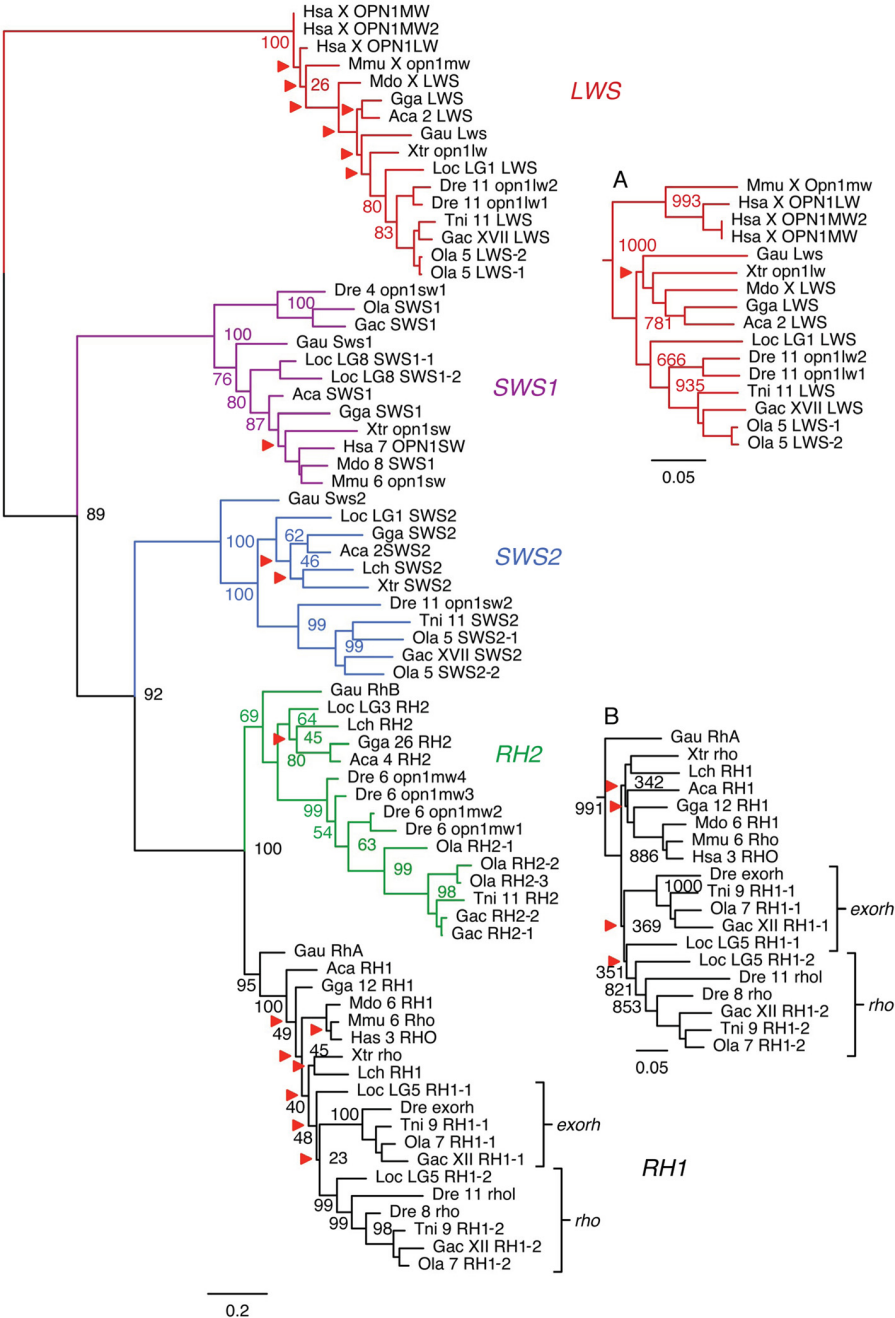
**Phylogenetic relationships between the visual opsin genes of the *LWS, SWS1, SWS2, RH1 *and *RH2 *clades.** Tree topology inferred with the phylogenetic maximum likelihood method from an amino acid sequence alignment, supported by a non-parametric bootstrap analysis with 100 replicates. Red arrowheads indicate nodes with bootstrap values lower than 50% that were not considered informative. The tree is rooted with the human *OPN3* sequence (not shown). Inlaid: **(A)** Neighbor joining (NJ) topology of the *LWS* clade, **(B)** NJ topology of the *RH1* clade. See Additional file 2: Figures S1 and S2 for full trees, including all bootstrap values and root. For the sequence names, species abbreviations are applied as described in *Methods*, followed by the number of the chromosome or linkage group where the gene is located (if known) and the gene/subtype name (see Table 1). Scale bars indicate phylogenetic distance as number of substitutions per site.

Genes for the five ancestral types of visual opsins present in the vertebrate ancestor (*LWS*, *SWS1*, *SWS2*, *RH2* and *RH1*) [4] were identified in sarcopterygians (including tetrapods and coelacanth) and actinopterygians (ray-finned fishes, including teleosts and spotted gar). These results are summarized in Table 1. In the phylogenetic analyses these sequences form five well-supported clusters: The *LWS* cluster forms a basal separate branch while the *SWS1*, *SWS2*, *RH2* and *RH1* cluster together with high support (Figure 1). This topology is supported by neighbor joining (NJ) and phylogenetic maximum likelihood (PhyML) methods (Additional file 2: Figures S1 and S2). The trees were rooted with the human *OPN3* sequence, as the *OPN3* gene has been shown to diverge before the diversification of the visual opsin genes [31,32]. Pinopsins and the vertebrate ancient (V/A) opsins often cluster with the visual opsins in phylogenetic analyses [5,31-33], and there are also related opsins in the tunicate *Ciona intestinalis* called *Ci-opsin1* and *Ci-opsin2*[34]. The overall topology presented in Figure 1 is not affected by the inclusion of these sequences in additional phylogenetic analyses (Additional file 2: Figures S3 and S4). All opsin phylogenetic tree files and the alignments they were made from are provided as a citable file set with a stable identifier – see reference [35].

**Table 1:** Summary of visual opsin sequences analyzed in this study

	**Species (genome assembly version)**	**Sequence names in phylogenetic analyses **^**a**^	**Type **^**b**^	**Chromosome, linkage group, or genomic scaffold**	**Loc. (Mb)**
**Sarcopterygians**					
Mammals	Human	Hsa X OPN1LW	LWS	X	153.41
	(GRCh37)	Hsa X OPN1MW2	LWS	X	153.49
		Hsa X OPN1MW	LWS	X	153.45
		Hsa 3 RHO	RH1	3	129.25
		Hsa 7 OPN1SW	SWS1	7	128.41
	Mouse	Mmu X Opn1mw	LWS	X	71.37
	(NCBIM37)	Mmu 6 Rho	RH1	6	115.88
		Mmu 6 Opn1sw	SWS1	6	29.33
	Grey short-tailed opossum	Mdo X LWS	LWS	X	14.66
	(MonDom5)	Mdo 6 RH1	RH1	6	246.61
		Mdo 8 SWS1	SWS1	8	188.61
Birds	Chicken	Gga LWS	LWS	^c^	
	(WASHUC2)	Gga 12 RH1	RH1	12	20.16
		Gga 26 RH2	RH2	26	4.38
		Gga SWS1	SWS1	^c^	
		Gga SWS2	SWS2	^c^	
Non-avian reptiles	Carolina anole lizard	Aca 2 LWS	LWS	2	88.66
	(AnoCar2.0)	Aca RH1	RH1	GL343273.1	0.64
		Aca 4 RH2	RH2	4	122.81
		Aca SWS1	SWS1	GL343828.1	0.11
		Aca 2 SWS2	SWS2	2	88.63
Amphibians	Western clawed frog	Xtr opn1lw	LWS	GL172911.1	0.21
	(JGI_4.2)	Xtr rho	RH1	GL172832.1	1.64
		Xtr opn1sw	SWS1	GL173116.1	0.61
		Xtr SWS2	SWS2	GL172911.1	0.22
Coelacanths	Coelacanth	Lch RH1	RH1	JH126975.1	0.80
	(LatCha1)	Lch RH2	RH2	JH126819.1	1.63
		Lch SWS2	SWS2	JH127263.1	0.54
**Actinopterygians**					
Holostean fishes	Spotted gar	Loc LG1 LWS	LWS	LG1	2.61
	(LepOcu1)	Loc LG5 RH1-1^d^	RH1	LG5	40.45
		Loc LG5 RH1-2	RH1	LG5	23.74
		Loc LG3 RH2	RH2	LG3	36.55
		Loc LG8 SWS1-1^e^	SWS1	LG8	10.39
		Loc LG8 SWS1-2^d^	SWS1	LG8	10.40
		Loc LG1 SWS2	SWS2	LG1	2.62
Teleost fishes	Zebrafish	Dre 11 opn1lw1	LWS	11	26.41
	(Zv9)	Dre 11 opn1lw2	LWS	11	26.41
		Dre exorh	RH1	Zv9_NA986	0.130 Kb
		Dre 8 rho	RH1	8	55.71
		Dre 11 rhol	RH1	11	19.52
		Dre 6 opn1mw1	RH2	6	41.11
		Dre 6 opn1mw2	RH2	6	41.12
		Dre 6 opn1mw3	RH2	6	41.12
		Dre 6 opn1mw4	RH2	6	41.13
		Dre 4 opn1sw1	SWS1	4	12.64
		Dre 11 opn1sw2	SWS2	11	26.41
	Three-spined stickleback	Gac XVII LWS	LWS	XVII	10.63
	(BROADS1)	Gac XII RH1-1^d,e^	RH1	XII	1.18
		Gac XII RH1-2	RH1	XII	0.81
		Gac RH2-1	RH2	scaffold 27	4.15
		Gac RH2-2	RH2	scaffold 27	4.16
		Gac SWS1	SWS1	scaffold 90	0.46
		Gac XVII SWS2	SWS2	XVII	10.62
	Medaka	Ola 5 LWS-1	LWS	5	27.02
	(MEDAKA1)	Ola 5 LWS-2	LWS	5	27.01
		Ola 7 RH1-1	RH1	7	17.43
		Ola 7 RH1-2	RH1	7	17.10
		Ola RH2-1	RH2	ultracontig 62	1.61
		Ola RH2-2	RH2	ultracontig 62	1.62
		Ola RH2-3	RH2	ultracontig 62	1.62
		Ola SWS1	SWS1	scaffold 1021	0.04
		Ola 5 SWS2-1	SWS2	5	27.01
		Ola 5 SWS2-2	SWS2	5	27.00
	Spotted green pufferfish	Tni 11 LWS	LWS	11	10.12
	(TETRAODON8)	Tni 9 RH1-1	RH1	9	6.68
		Tni 9 RH1-2	RH1	9	6.48
		Tni 11 RH2	RH2	11	5.97
		Tni 11 SWS2	SWS2	11	10.12
**Cyclostomes**					
Lampreys	Pouched lamprey	Gau Lws	LWS		
		Gau RhA	RH1		
		Gau RhB	RH2		
		Gau Sws1	SWS1		
		Gau Sws2	SWS2		

^a^For the sequence names, species abbreviations are applied as described in *Methods*, followed by the number of the chromosome or linkage group where the gene is located (if known) and the gene/subtype name. Where available, gene names follow approved symbols by the HUGO Gene Nomenclature Committee and interacting committees, accessed via *http://www.genenames.org*, and the Zebrafish Model Organism Database (ZFIN), accessed via *http://www.zfin.org*. *Geotria australis* gene names follow [36].

^b^Subtype designations follow the tree topology presented in Figure 1.

^c^In the chicken genome assembly available in the Ensembl genome browser (WASHUC2) only two of the five ancestral visual opsin types were identified. However the three missing genes have previously been cloned and are available in the NCBI GenBank database (see Additional file 1).

^d^Sequences manually predicted *de novo* (see *Methods*).

^e^Sequence predictions are fragments and do not span the whole length of the alignment.

### Visual opsin gene repertoires

The repertoires of visual opsins in mammals and birds have been described in detail in numerous previous studies, and the anole lizard (*Anolis carolinensis*) opsin genes have been described more recently [37]. These data are consistent with our analyses (Figure 1), which confirm that the ancestral repertoire in jawed vertebrates consisted of five visual opsin genes with losses of *SWS2* and *RH2* in mammals. The Western clawed frog (*Xenopus tropicalis*) also seems to have lost the *RH2* gene, although this could be due to gaps in the genome assembly. Three full-length and one fragmented visual opsin gene sequences were identified in the coelacanth (*Latimeria chalumnae*) genome assembly. The full-length sequences cluster within the *RH1, RH2* and *SWS2* branches (Figure 1). The *RH1* and *RH2* sequences have previously been reported [38]. The presence of only three visual opsin sequences indicates that there seem to have been losses of visual opsin genes in this lineage. The fragmented gene sequence appears to be a pseudogene sharing sequence similarity to known *SWS1* sequences, but with a premature stop-codon and a frame-shift mutation within the first exon. A sequence fragment bearing the same mutations at the same locations has been identified previously [38] as one of two non-overlapping clones postulated to be parts of an *SWS1* pseudogene. However, the second clone reported by these authors, instead corresponds to a fragment of the full-length *SWS2* sequence that we identified. All five ancestral types of vertebrate visual opsin genes are present in all the investigated actinopterygian genomes (including spotted gar and teleosts), except the spotted green pufferfish, where an *SWS1* sequence could not be identified (Figure 1). Additionally, *LWS, SWS2, RH1* and *RH2* sequences often occur as multiple local duplicates in teleosts (Table 1). In the spotted gar genome assembly we could identify seven visual opsin genes: one gene each of the *LWS*, *SWS2* and *RH2* types and two genes each of the *SWS1* and *RH1* types (Figure 1). The two *SWS1* genes are located adjacent to each other on the same linkage group (LG8) approximately 8 Kb apart (see Table 1), and are thus most likely the result of a local duplication. The two *RH1* genes are located on the same linkage group (LG5) approximately 16.6 Mb apart, one with introns (*RH1-1* in Table 1 and Figure 1) and one without (*RH1-2*). These duplicate *RH1* genes on the same chromosome have previously been identified in teleost fish species: the one with introns, called exo-rhodopsin, is expressed outside of the retina; and the one without introns, called rhodopsin, is expressed in rods [39]. The intron-less rhodopsin gene is the result of a retrotranscription event [40]. We could identify these duplicate *RH1* sequences in all investigated teleost genomes (*RH1-1* and *RH1-2* in Table 1 and Figure 1). Additionally, the zebrafish has two copies of the intron-less rhodopsin gene, located on two different chromosomes. These zebrafish genes have previously been called *rh1* and *rh1-2*[41] or *rho* and *rhol* (for *rhodopsin-like*) (Table 1). The teleost exo-rhodopsins form a well-supported cluster in the phylogenetic analyses (marked *exorh* in Figure 1) while the putative spotted gar exo-rhodopsin (*RH1-1*) has an uncertain position within the RH1 cluster. On the other hand, the rhodopsin gene of the spotted gar clusters together with the teleost rhodopsin genes with high support, forming a well-defined actinopterygian cluster (marked *rho* in Figure 1).

Five visual opsins have previously been cloned and characterized from the pouched lamprey (*Geotria australis*) [36] and were therefore included in our phylogenetic analyses to provide relative dating. In line with previous analyses [2,7,36] the pouched lamprey *LWS, SWS1* and *SWS2* sequences cluster with high support within their respective branches (Figure 1). We also find that the sequences called *RhA* and *RhB* represent the *RH1* and *RH2* genes, with high support in all our phylogenetic analyses (Figure 1, Additional file 2). Thus it is likely that all five visual opsin genes were present before the divergence of cyclostomes, such as the lampreys, and jawed vertebrates.

### Phylogenetic analysis of the oxytocin and vasopressin receptor genes

**Figure 2:**
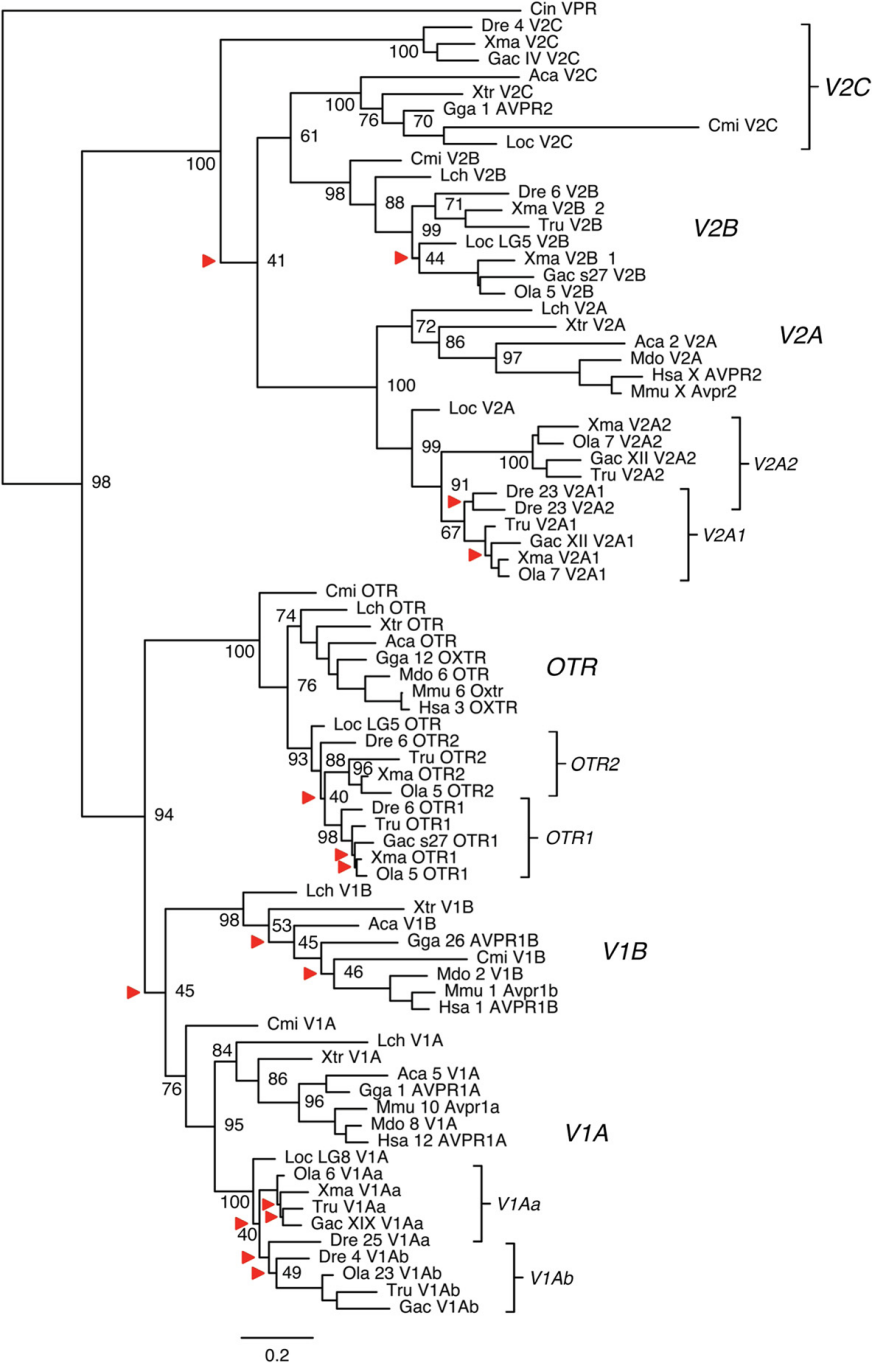
**Phylogenetic relationships between oxytocin and vasopressin receptor subtype genes.** Tree topology inferred with the phylogenetic maximum likelihood method from an amino acid sequence alignment, supported by a non-parametric bootstrap analysis with 100 replicates. Arrowheads indicate nodes with bootstrap values lower than 50% that were not considered informative. Rooted with the common octopus *OTR, CTR1* and *CTR2* sequences (not shown). See Additional file 2: Figures S5 and S6 for full trees with all bootstrap values and root, including a neighbor joining topology. Sequence names and scale as in as in Figure 1. Teleost fish duplicates are indicated by brackets. The V2C sequences do not form a well-supported clade; this is also indicated with a bracket. Approved gene names are used for human, mouse and chicken genes, otherwise subtype names are used. Some of the sequence predictions used to make the tree are fragments and do not span the whole length of the alignment (see Additional file 1).

We have updated recent phylogenetic analyses of the OT/VP-R gene family [29,30] by including sequences from the coelacanth, spotted gar and Southern platyfish. This was done in order to improve the taxonomic representation and complement our conserved synteny analysis, which includes positional data from the spotted gar genome assembly. Both NJ and PhyML trees were made (Additional file 2: Figures S5 and S6), and the PhyML tree is shown in Figure 2. Our updated phylogenetic analyses are consistent with the previously cited literature, but also show several new results.

The clusters for oxytocin receptors (OTR) and V1-type vasopressin receptors (V1A, V1B) are well-supported, and the tree shows that the spotted gar, like teleosts, lacks the *V1B* subtype while the coelacanth has all three (Figure 2). With regard to V2-type vasopressin receptors, *Yamaguchi et al (2012)* were able to define three types by using synteny data [29]. Our phylogenetic analyses and the analysis of conserved synteny described below are consistent with three ancestral V2-types, and we propose the nomenclature *V2A*, *V2B* and *V2C* for these receptors. As in the previously cited analyses, the *V2C* receptors form two paraphyletic branches, with teleost *V2C* sequences clustering basal to the other V2-type branches (Figure 2), likely due to a faster rate of sequence evolution. However, their chromosomal locations support their orthology with tetrapod and spotted gar *V2C* sequences (see below). The coelacanth lacks a *V2C* sequence but has both *V2A* and *V2B,* making it the only analyzed species in the sarcopterygian lineage (which also includes tetrapods) with a *V2B* gene*.* Since *V2C* sequences were found in the Western clawed frog, anole lizard and chicken genomes (Figure 2), the loss in the coelacanth is likely lineage-specific. Taken together with the spotted gar, which has all three V2-type sequences (Figure 2), this indicates that the *V2A, V2B* and *V2C* subtypes arose early in vertebrate evolution, although there have been several differential losses in different vertebrate classes. Sequences from the Southern platyfish were included since this species was found to have the *V2C* sequence that previously had only been found in zebrafish and three-spined stickleback [30]. These three species do not form a monophyletic group within teleosts, which indicates that *V2C* genes could have been lost several times in teleost evolution. The Southern platyfish is also the only teleost where duplicate *V2B* sequences were found (*V2B-1* and *V2B-2* in Figure 2).

Database identifiers, location data, genome assembly information, and annotation notes for all identified OT/VP-R sequences are included in Additional file 1. The OT/VP-R phylogenetic tree files and the alignment they were made from are provided as a citable file set with a stable identifier – see reference [42].

### Phylogenetic analysis of the L-type voltage-gated calcium channel alpha subunits

Four genes of the CACNA1-L family were identified in the tetrapod genomes investigated: *CACNA1D*, *CACNA1F*, *CACNA1C* and *CACNA1S.* However *CACNA1F* could not be identified in the chicken genome assembly or in any other avian genome available. The Western clawed frog was excluded from the analysis because the short scaffolds of the genome assembly (JGI4_1) did not allow complete gene sequences to be identified. In the teleost genomes investigated all four genes are present, with additional putative 3R duplicates of the *CACNA1D, -1F* and *-1S* genes in all four teleost genomes. These results are detailed in Additional file 1, with database identifiers, location data, genome assembly information, and annotation notes for all identified sequences.

The *CACNA1F*, *-1C* and *-1S* genes form three distinct clusters in the bootstrapped NJ (Additional file 2: Figure S7) and PhyML (Figure S8) analyses, while the putative teleost *CACNA1D* subtype cluster is only resolved in the NJ tree (Additional file 2: Figure S7). These results are presented in Additional file 2. All CACNA1-L phylogenetic tree files and the alignment they were made from are provided as a citable file set with a stable identifier – see reference [43].

### Conserved synteny analysis

In total, 41 neighboring gene families showed patterns of conserved synteny in the chromosomal regions harboring members of the five main gene families. Seven of the 41 gene families were discarded upon preliminary analyses because their multitude of members in the genome databases made phylogenetic analyses unreliable, or because their topologies could not be resolved, leaving 34 gene families in our final dataset. These families are summarized in Table 2. The locations of the identified genes were recorded for 12 species, representing five of the vertebrate classes, with available genome assemblies (see *Conserved synteny analysis* in *Methods*), and phylogenetic analyses using neighbor joining (NJ) and phylogenetic maximum likelihood (PhyML) methods were carried out for each gene family in order to determine orthology and paralogy relationships. Database identifiers, location data and annotation notes for the neighboring gene families, including those that were discarded, are provided in Additional file 3 and all corresponding alignments and phylogenetic trees are provided as a citable file set with a stable identifier – see reference [44].

**Table 2:** Neighboring gene families analyzed for conserved synteny

**Symbol**	**Description**	**Root**^**1**^
ATP2B	*ATPase, Ca++ transporting, plama membrane*	
B4GALNT	*Beta-1,4-N-acetyl-galactosaminyl transferase*	*Midpoint*
CACNA2D	*Calcium channel, voltage-dependent, alpha 2/delta subunit*	
CAMK1	*Calcium/calmodulin dependent protein kinase*	
CDK	*Cyclin-dependent kinase, members 16, 17 and 18*	*C. elegans*
CELSR	*Cadherin, EGF LAG seven-pass G-type receptor (flamingo homolog, Drosophila)*	
CNTN	*Contactin precursor*	
COPG	*Coatomer protein complex, subunit gamma*	
ERC	*ELKS/RAB6-interacting/CAST family*	
FLN	*Filamin*	
GXYLT	*Glucoside xylosyltransferase*	
IKBKE	*Kinase epsilon and TANK-binding kinase*	
IQSEC	*IQ motif and Sec7 domain containing*	
KDM	*Lysine specific demethylase 5*	
KLHDC	*Kelch domain containing 8*	*C. intestinalis*
L1CAM	*L1 cell adhesion molecule*	
LRRN	*Leucine rich repeat neuronal*	*C. intestinalis*
MAGI	*Membrane associated guanylate kinase, WW and PDZ domain containing*	
PHTF	*Putative homeodomain transcription factor*	*C. savignyi*
PLG	*Plasminogen ortholog*	*C. intestinalis*
PLXNA	*Plexin A*	
PPM1	*Protein phosphatase, Mg2+/Mn2+ dependent*	
PRICKLE	*Prickle homolog*	
PTPN	*Protein tyrosine phosphatase, non-receptor type*	
RBM	*RNA binding motif protein*	
RSBN	*Round spermatid basic protein*	
SEMA3	*Sema domain, immunoglobulin domain (Ig), short basic domain, secreted, (semaphorin)*	*Midpoint*
SRGAP	*SLIT-ROBO Rho GTPase activating protein*	*C.intestinalis*
SYP	*Synaptophysin*	*C.elegans*
TIMM17	*Translocase of inner mitochondrial membrane 17*	*C.elegans*
TWF	*Twinfilin*	
UBA	*Ubiquitin-like modifier activating enzyme, members 1 and 7*	
USP	*Ubiquitin specific peptidase, members 4, 11, 15 and 19*	*Midpoint*
WNK	*WNK lysine deficient protein kinase*	

^1^ If other than *Drosophila melanogaster.*

For the majority of the identified neighboring gene families, 23 out of 34, both the NJ trees and the PhyML trees support duplications in the same time-window as the 2R events, i.e. after the divergence of invertebrate chordates and vertebrates, but before the divergence of sarcopterygians and actinopterygians. Several of the 34 identified families also have duplicate teleost branches supporting duplications in the same time-window as the teleost-specific 3R event. Four additional gene families are consistent with duplications in 2R, but lack invertebrate family members to date the duplications. The remaining seven gene families have some subtype clusters that are unresolved, or an unresolved branching of the identified tunicate or lancelet sequences, which makes them inconclusive with regard to the duplications in 2R. Detailed notes on the tree topologies for the neighboring gene families are provided in Additional file 4.

**Figure 3:**
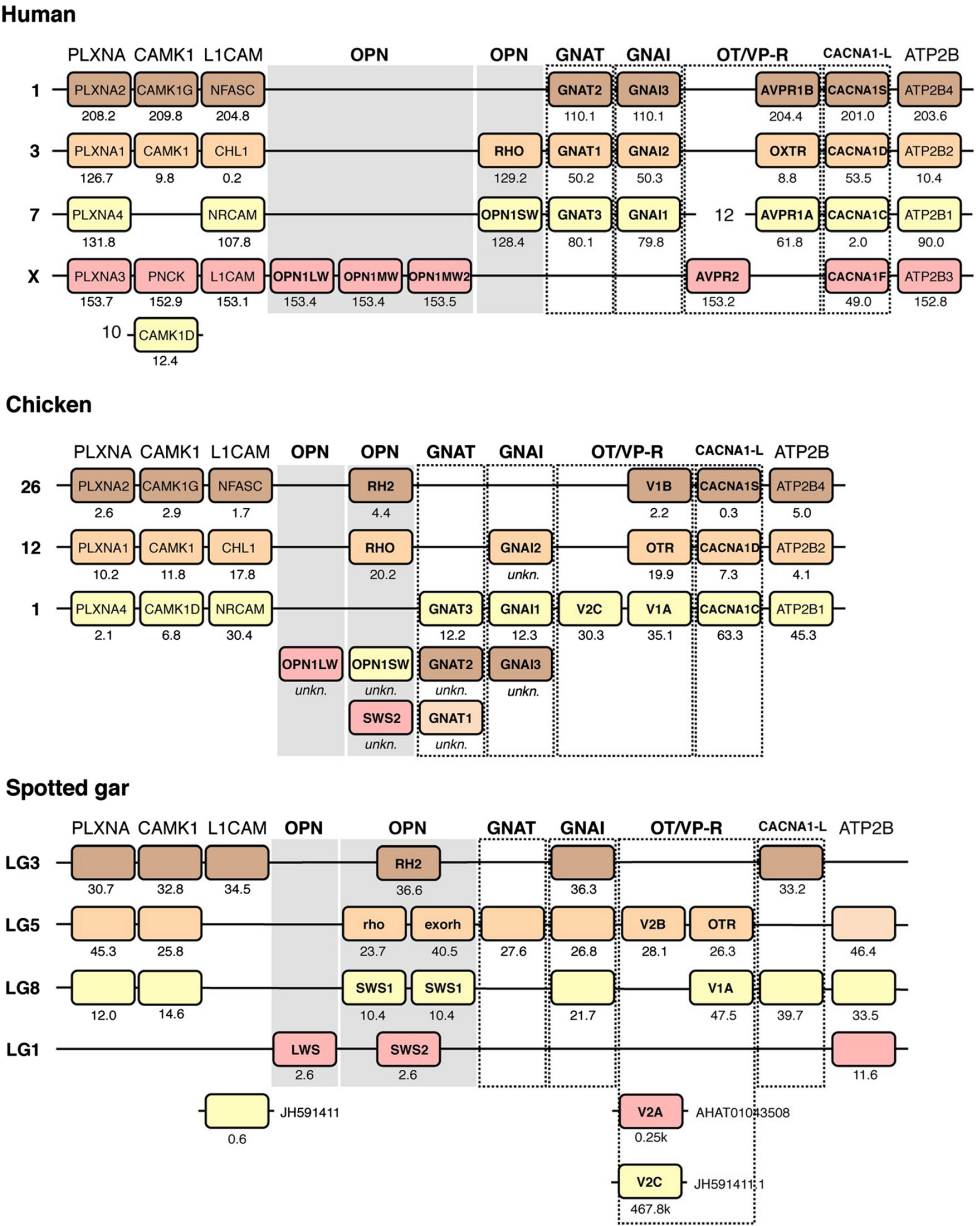
**Conserved synteny between 2R-generated chromosome blocks.** The identified paralogous chromosomal regions in the human, chicken and spotted gar genomes. In addition to the main gene families, the neighboring gene families with a full quartet of paralogous genes (ATP2B1, CAMK1, L1CAM and PLXNA) are included for reference. Colors are applied following the human and chicken chromosomes in order to show conserved synteny as well as sequence homology between species. Four paralogous regions can be observed in the human and spotted gar genomes. In the chicken genome the orthologs of the human genes on chromosome X could not be identified in the genome assembly. Orthologs for the genes on human chromosomes 7 and 12 are located on chromosome 1 in chicken and LG8 in spotted gar, indicating a split of this region in the human lineage. Several chicken genes have not been mapped to any chromosomal location. Their sequences for phylogenetic analyses were retrieved from NCBI. To facilitate comparisons between species, the names of the human orthologs have been applied to the chicken genes except for the visual opsin and OT/VP-R families where the gene names used in Figures 1 and 2 are applied. Note that the human and chicken *V2* receptor sequences correspond to different subtypes: *V2A* and *V2C* respectively (Figure 2).

To investigate the involvement of the whole genome duplications, the chromosomal locations of all neighboring family members were recorded and compared across species. A compiled list of comparisons between the human, chicken, zebrafish and three-spined stickleback chromosome regions is shown in Additional file 5. This dataset shows the conserved synteny between the visual opsin gene-bearing chromosome regions in the analyzed genomes, which also includes the OT/VP-R, CACNA1-L and GNAT/GNAI gene families. These paralogous chromosome regions correspond to blocks present on human chromosomes 1, 3, 7, 12 and X, and chicken chromosomes 1, 12 and 26 (Figure 3). The blocks on human chromosomes 7 and 12 seem to be the result of a linkage break in the human lineage, since the chicken orthologs of the genes in both of these blocks are all located on chicken chromosome 1. This is supported by our orthology analysis of the spotted gar genome (Additional file 5). Notably, no chicken orthologs of the genes located on the human X chromosome were found in the chicken genome database, indicating that this whole segment of the chicken genome is missing from the genome assembly (WASHUC2, latest accessed on Ensembl 71, Apr 2013).

**Figure 4:**
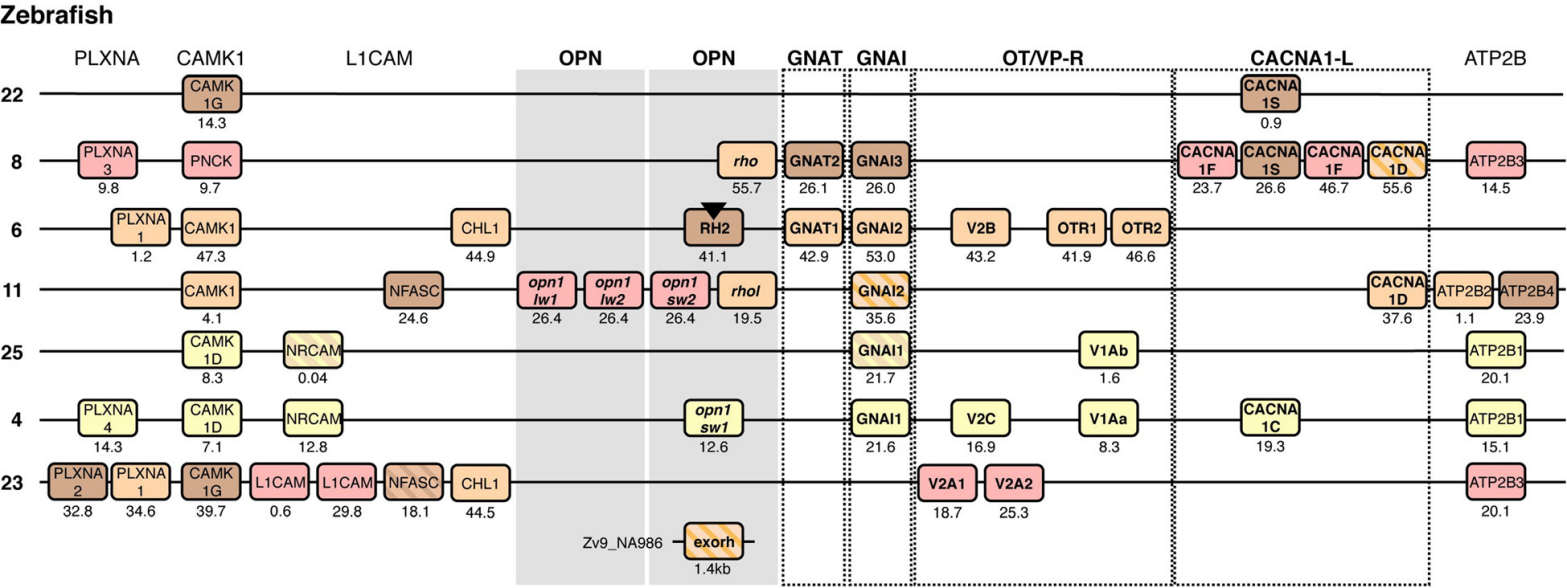
**Paralogous chromosome blocks in the zebrafish genome.** Gene families and colors applied as in Figure 3. Dashed boxes indicate divergences between the NJ tree and PhyML tree topologies. The color of the dashes nonetheless indicate the likely homology relationships. Orthologs of the human genes located on chromosomes 1, 3 and X are intermingled on zebrafish chromosomes 22, 8, 6, 11 and 23, indicating major rearrangements. In addition genes located on human chromosomes 7 and 12 and chicken chromosome 1 have orthologs on zebrafish chromosomes 25 and 4, indicating 3R-generated chromosome blocks. The black arrowhead marks the four local duplicates of the *RH2* gene in the zebrafish genome (see Table 1). The full conserved synteny analysis, including all gene families and three-spined stickleback, is included in Additional file 5.

The teleost-specific duplication of whole chromosome regions is evident in the teleost genomes for the regions harboring *OPN1SW*, *V1A*, *CACNA1C* and *GNAI1* genes (Figure 4). These regions of chicken chromosome 1 and human chromosomes 7 and 12 correspond to blocks on zebrafish chromosomes 4 and 25 (Figure 4), and three-spined stickleback linkage groups IV and XIX (Additional file 5), with several of the gene families having teleost-specific duplicates in both chromosome blocks. However, our analyses also demonstrate that there have been extensive rearrangements in the teleost genomes, which obscure the involvement of both the 2R and 3R events. For instance, genes located on the chromosomes 1 and 3 and the X chromosome in the human genome have orthologs distributed between zebrafish chromosomes 6, 8, 11 and 23 in a way that suggests translocation of paralogous genes between these chromosome regions after 3R (Figure 4 and Additional file 5).

In order to date the translocations seen in the teleost genomes better, orthology predictions between the human neighboring family members and the spotted gar genome were made (see *Methods*). The locations of the predicted spotted gar orthologs were then recorded and compared with the human, chicken, zebrafish and three-spined stickleback chromosome regions (Additional file 5). While only around 75% of putative spotted gar orthologs could be found for the human neighboring gene family members, this analysis of conserved synteny between the human and spotted gar shows no translocations in the spotted gar genome. With a few exceptions, the identified putative spotted gar orthologs are located on linkage groups 1, 3 and 5, which correspond to the regions on human chromosomes X, 1 and 3 respectively, and linkage group 8 which corresponds to the regions on human chromosomes 7 and 12 (Figure 3).

## Discussion

In order to investigate the evolution of the visual opsin genes related to the two rounds of early vertebrate whole genome duplications, 2R, we have analyzed several neighboring gene families identified in the same chromosomal regions as the human visual opsin genes. Specifically, we investigated whether there were other gene families showing conserved synteny with these and whether they underwent gene duplications in the same time-window as the 2R events. During the process of these analyses we realized that the chromosomal regions of the visual opsin genes overlapped with similar ongoing analyses in our laboratory of the oxytocin and vasopressin receptor gene family (OT/VP-R), the G-protein alpha transducing subunit (GNAT) and G-Protein alpha inhibiting subunit (GNAI) gene families, as well as the gene family of L-type voltage-gated calcium channel alpha subunits (CACNA1-L). These gene families are referred to as the “main” gene families in our analyses. Their evolution will be discussed below in conjunction with the evolution of the visual opsins.

### The chromosomal regions harboring the visual opsin genes were duplicated in the vertebrate whole genome duplications

**Figure 5:**
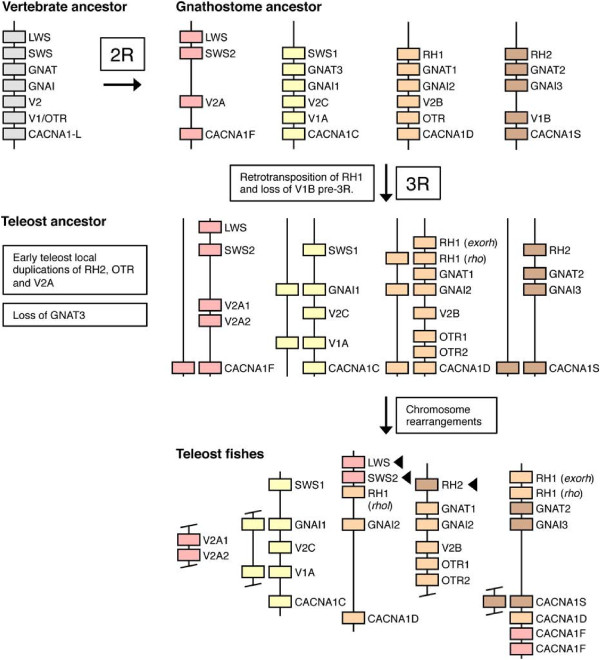
**Proposed evolutionary history of the visual opsin gene-bearing chromosome regions.** The proposed evolutionary scenario also includes the oxytocin/vasopressin receptor gene family (OT/VP-R), the voltage-gated calcium channel L-type alpha subunit gene family (CACNA1-L) and the G-protein alpha transducing (GNAT) and alpha inhibiting (GNAI) gene families. This scenario is consistent with data from additional neighboring gene families (see *Conserved synteny analyses* in *Results*). Local duplications before 2R occurred in the visual opsin and OT/VP-R gene families, giving rise to ancestral *SWS* and *LWS* genes, and ancestral *V1/OTR* and *V2* genes respectively. The chromosome region subsequently quadrupled in 2R, giving rise to paralogous genes in all gene families. For the visual opsin gene family, the ancestral *SWS* gene gave rise to the *SWS1, SWS2, RH1* and *RH2* genes. However, only one copy of the *LWS* gene was retained. Early in actinopterygian evolution, before the divergence of spotted gar and teleost fishes, the *RH1* gene was retrotransposed, giving rise to an intron-less *RH1* duplicate. In the OT/VP-R family the *V1B* gene was lost. Following this, the chromosome regions duplicated in 3R, giving rise to duplicates of *GNAI1, GNAI2, V1A, CACNA1D, CACNA1C* and likely also *RH1* (*rho* and *rhol*) and CACNA1F. We propose the nomenclature *V1Aa* and *V1Ab* for the 3R-generated *V1A* duplicates. After 3R, local duplications of the *RH2, OTR* and *V2A* genes occurred and extensive chromosomal rearrangements moved genes between the paralogous chromosome regions. Black arrowheads mark *LWS, SWS1* and *RH2* genes that have lineage-specific local duplicates in some teleost species.

In total, the chromosomal locations and phylogenetic analyses of 34 neighboring gene families have been analyzed. The orthology and paralogy relationships within each family were inferred and, using relative dating, the time-window for the expansion of the gene families was determined. The synteny data and phylogenetic analyses taken together show that the chromosome regions bearing the visual opsin genes are paralogous and were formed by chromosome duplications in the same time-window as the 2R events, after the divergence of invertebrate chordates and before the divergence of actinopterygians (including teleosts and spotted gar) and sarcopterygians (including tetrapods and coelacanth). Our analyses also show that the chromosomal regions were duplicated once more in early teleost evolution, consistent with the time-window of the 3R event. Our proposed evolutionary scenario for the evolution of these chromosome regions is presented in Figure 5. In the human genome, the paralogous chromosomal regions correspond to blocks of chromosomes 1, 3, 7, 12 and X (Figure 3), with the blocks on 7 and 12 together representing one of the four ancestral blocks. These genomic regions have previously been identified as part of a paralogon in large-scale genomic analyses [10,11,45].

### Chromosomal rearrangements in teleost genomes, but not in the spotted gar genome

In the teleost genomes the investigated orthologous genes of the human genes located on chromosomes 1, 3 and X seem to have been rearranged so that the genes are intermingled. In zebrafish this involves chromosomes 8, 23, 11 and 6 (Figure 4) and in the three-spined stickleback linkage groups XII, XVII and scaffold 27 (Additional file 5). These major rearrangements are in line with previous whole genome analyses [10,45]. Recently *Amores et al.* published an analysis of the genome of the spotted gar (*Lepisosteus oculatus*), which belongs to a actinopterygian lineage that diverged before 3R and thus should not have the same rearrangements [46]. They found that the spotted gar seemed to have fewer rearrangements in general compared to the teleosts and that the synteny was more conserved between human and spotted gar than between spotted gar and zebrafish due to post-3R rearrangements in the teleost lineage. To see if this was the case for the regions housing the visual opsin genes, we performed an orthology prediction between human and spotted gar protein predictions and recorded the chromosomal locations of the putative spotted gar orthologs (Additional file 5). This is summarized in Figure 3. By comparing the chromosomal regions of human, chicken, spotted gar, zebrafish and three-spined stickleback we found that the synteny is indeed more conserved between human, chicken and spotted gar than between spotted gar and the teleosts, in agreement with the global genome analyses [46]. This further supports the notion that 3R contributed to the major rearrangements seen in teleost genomes [10,45].

### Evolution of the visual opsin gene family

**Figure 6:**
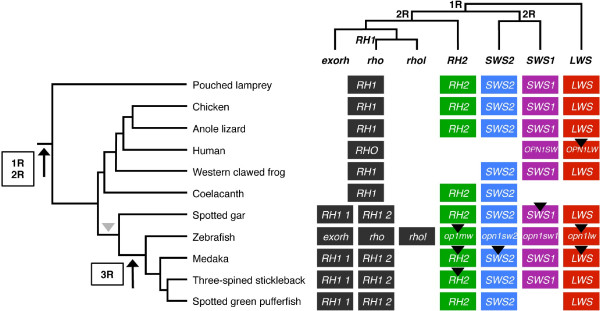
**Visual opsin gene repertoires in vertebrates.** The tree to the left shows the evolutionary relationship between species used in our analyses, with the time-windows for the 2R and 3R events. The upper tree shows the relationship between *LWS, SWS1, SWS2, RH1* and *RH2* visual opsins inferred from phylogenetic analyses and synteny data. Black arrowheads mark the presence of local duplicates. The retrotransposition event that gave rise to intron-less rhodopsin (*RH1*) genes in spotted gar and teleosts is marked with a grey arrowhead in the left panel. The intron-less *RH1* genes are called *rho* and *rhol*, and *exorh* denotes the exo-rhodopsin genes, using the approved zebrafish names. For human and zebrafish genes, the approved gene names are used as indicated in footnote a of Table 1.

Our phylogenetic analyses and the analyses of conserved synteny of the visual opsins and their chromosomal regions support the evolutionary scenario previously proposed by us [22,23] where a local duplication occurred before 2R giving rise to two ancestral genes, *SWS* and *LWS*. These genes later duplicated in 2R as part of a larger chromosomal block so that the ancestral *SWS* gene gave rise to the types *SWS1*, *SWS2*, *RH1* and *RH2* and the ancestral *LWS* gave rise to four copies out of which only one has been retained, namely the *LWS* type (Figure 5). Although the pinopsins and vertebrate ancient (V/A) opsins cluster with the visual opsins in phylogenetic trees (Additional file 2: Figures S3 and S4), the present synteny analysis does not indicate that pinopsins and V/A opsins arose in the same chromosome duplications that gave rise to the visual opsin gene family. The current repertoire of visual opsin genes in vertebrates, and their evolution, is summarized in Figure 6.

Five of the seven identified spotted gar genes represent the *LWS*, *SWS1*, *SWS2* and *RH2* types of visual opsin genes, while the other two represent the *RH1* type (Figure 1): one intron-containing (RH1-1) and one intron-less (RH1-2). The intron-containing *RH1* gene is likely the ortholog of the teleost exo-rhodopsin gene (*exorh*), named for its expression mainly in the pineal complex of the teleost brain instead of the retina [39]. The intron-less *RH1* gene is likely the ortholog of the teleost rhodopsin (*rho*) gene, a retrotranscript of the exo-rhodopsin gene [47]. Our analyses indicate that the retrotransposition event occurred before the divergence of holostean fishes (including gars) and teleosts (Figure 6). The intron-less spotted gar *RH1* is presumably the ortholog of a gene that has previously been identified in the longnose gar (*Lepisosteus osseus*) as rhodopsin [48]. In the zebrafish there is an additional intron-less *RH1* gene called *rhol* for rhodopsin-like (Figure 2), located on a different chromosome than the *rho* gene (Table 1). *Morrow et al.,* who identified the two intronless *RH1* genes in the zebrafish genome, also identified similar duplicated genes in a few other cyprinid species [41]. Analyses performed by other researchers have shown that duplicated intron-less *RH1* genes seem to be present in some non-cyprinid teleost species as well, including the deep-sea dwelling short-fin pearl-eye [49] and the Japanese and European eels [50,51]. This suggests that the two intron-less *RH1* genes might have originated in 3R, following the retrotransposition event. However, to confirm that *rho* and *rhol* are 3R duplicates, information about chromosomal locations is needed from several species. The presence of two intronless *RH1* genes in the above-mentioned species, but not in the medaka, spotted green pufferfish and three-spined stickleback, suggests loss in the latter species or in their common ancestor. In the zebrafish, the regions harbouring the duplicated *RH1* genes *rho* and *rhol* on chromosome 8 and 11 have undergone major rearrangements. Our analyses show that these chromosomes contain paralogous members of the CACNA1-L, ERC, CACNA2D, PRICKLE and MAGI families (see Additional file 5). The CACNA2D and MAGI tree topologies support a 3R duplication of these paralogous gene copies. However, the paralogous copies in the trees of the CACNA1-L, ERC and PRICKLE families are inconsistent. While it is a likely scenario, we cannot say for certain that the duplicate intron-less *RH1* genes originated in 3R.

### Evolution of the GNAT and GNAI gene families

In our previous analyses of the GNAT gene family we concluded that the GNAT-GNAI gene pairs seen today in many vertebrates had an origin in a local duplication preceding 2R. The 2R events subsequently gave rise to the *GNAT1-GNAI2*, *GNAT2-GNAI3* and *GNAT3-GNAI1* gene pairs [21]. This is in line with previous studies from other research groups, see for instance [27]. Consequently, independent losses of the *GNAT3* gene have occurred in the teleost and amphibian lineages and possibly the sea lamprey (*Petromyzon marinus*) lineage after 2R [27,28]. We identified putative 3R duplicates in the GNAI gene family for the *GNAI1* and *GNAI2* genes in our previous analysis [21], although we had no synteny data to support the hypothesis. In the present analyses 10 gene families have putative 3R duplicates located on the *GNAI1*-bearing chromosomes in zebrafish (chromosomes 4 and 25). Six of these families show a clear 3R topology in both phylogenetic analyses. In the three-spined stickleback 12 neighboring gene families have members on the *GNAI1*-bearing chromosomes (IV and XIX), seven of which show a clear 3R topology in both phylogenetic analyses. In addition, three of the 10 families in zebrafish as well as three out of 12 families in three-spined stickleback show a topology supporting 3R in either the NJ or PhyML tree. For the *GNAI2* genes, four neighboring gene families have putative 3R duplicates on the *GNAI2* bearing chromosomes in zebrafish (chromosomes 6 and 11). Three of these show a clear 3R duplication pattern. In three-spined stickleback only one family has members on the *GNAI2* bearing chromosome and scaffold (XVII and scaffold 27), although its topology is unclear with regard to 3R. These results are summarized in Additional file 5. Our orthology predictions between human and spotted gar identified a single putative *GNAT1* ortholog as well as a single putative *GNAI1-3* ortholog (Figure 3). Taken together this analysis supports a possible 3R expansion of the *GNAI1* and *GNAI2* genes and their chromosomal regions. This also corroborates the loss of the *GNAT3* gene in the teleost and amphibian lineages independently.

### Evolution of the oxytocin and vasopressin receptor gene family

Our phylogenetic analysis of the OT/VP-R family (Figure 2) shows that the vertebrate gene family consists of six ancestral members, *OTR, V1A, V1B, V2A, V2B* and *V2C*. We propose a simplified nomenclature for the V2-type receptors based on the evolutionary scenario below. The *V2A* receptors form the largest branch and include the well-characterized mammalian V2 receptor encoded by the *AVPR2* gene. The *V2B* receptor subtype was identified independently by us [30] and other researchers [29], and includes mostly actinopterygian sequences as well as coelacanth and elephant shark. The *V2C* receptor subtype was first reported by us as *V2-like* in zebrafish and three-spined stickleback [30], and it was later characterized as *V2bR2* by *Yamaguchi et al.*[29]*.* Unlike *V2B*, the *V2C* branch includes frog and lizard sequences, as well as the known chicken V2 receptor first characterized as VT1 [52]. Like the receptors OTR, V1A and V1B, both *V2B* and *V2C* seem to signal via the DAG/IP3/Ca^2+^ pathway, while the adenylyl cyclase/PKA/cAMP signaling typical for *V2A* receptors seems to constitute an evolutionary switch in the OT/VP-R family [29].

Our current phylogenetic analysis and the analyses of the visual opsin gene-bearing chromosome regions allowed us to deduce the following evolutionary scenario for the OT/VP-R gene family: Two ancestral genes were present on the same ancestral vertebrate chromosome before 2R, one giving rise to *V1A*, *V1B* and *OTR* through 2R and one giving rise to *V2*A, *V2B* and *V2C* (Figure 5). The ancestral linkage is still conserved with *V2B* and *OTR* genes located together, and *V1A* and *V2C* genes located together on the same chromosomes. In the teleost lineage, the 3R event gave rise to two copies of *V1A,* called *V1Aa* and *V1Ab,* as part of the same chromosome regions as the teleost *GNAI1* genes (Figure 4). The phylogenetic analysis (Figure 2) is consistent with this, although the *V1Aa* and *V1A*b sequences do not form two well-supported clusters. In the teleosts we also identified local duplicates of *OTR*, called *OTR1* and *OTR2*, and of *V2A*, called *V2A1* and *V2A2*.

### Evolution of the voltage-gated calcium channel, L-type gene family

The CACNA1-L gene family is one of three subfamilies of voltage-gated calcium channel alpha subunits. The CACNA1-L subfamily, which belongs to the paralogon described herein, has four members in mammals and up to seven members in teleost fish. A more comprehensive analysis of this gene region and the remaining two voltage-gated calcium channel families, located in other paralogons, is in progress (*Widmark et al.*).

The phylogenetic analyses (Additional file 2: Figures S7 and S8) as well as the analyses of conserved synteny performed in this study show that the CACNA1-L gene family expanded in 2R, before the radiation of vertebrates, with one ancestral gene giving rise to the *CACNA1S, CACNA1D, CACNA1C* and *CACNA1F* subtype genes. Subsequently the *CACNA1S*, *-1D* and *-1F* genes duplicated in 3R as part of the visual opsin gene-housing chromosome regions (Figure 5). As for several other gene families in this paralogon, these teleost duplicates have been translocated between the paralogous chromosome regions. For instance, teleost *CACNA1S, CACNA1D* and *CACNA1F* genes have all been translocated to zebrafish chromosome 8 (Figure 4) and stickleback linkage group XII (Additional file 5). As mentioned previously, we have observed similar rearrangements for other regions in teleost genomes during analyses of the somatostatin receptor gene regions [53]. The *CACNA1D* teleost cluster is not well resolved in the phylogenetic maximum likelihood analysis (Additional file 2: Figure S8), and no putative spotted gar sequence could be identified in the orthology predictions. However, our conserved synteny analyses as well as previous whole-genome analyses [10,45] are consistent with our conclusions.

### Implications for early vertebrate radiation

Our analyses show that the gnathostome opsin repertoire with the four subtypes *RH1*, *RH2*, *SWS1*, *SWS2* clearly resulted from a quadruplication of an ancestral chromosome block (that also included the adjacent but more distantly related *LWS* genes without surviving duplicates). The presence of orthologs of all these genes in the pouched lamprey (*Geotria australis*) [7] imply that these chromosome-based gene duplications took place before the lamprey-gnathostome divergence, as previously proposed [54]. Our present extensive characterization of these paralogous gene regions in a broad range of vertebrates demonstrates that they resulted from the quadruplication of a very large chromosomal block in the time window of 2R. Thus, it would follow that the lamprey lineage diverged after the two basal vertebrate tetraploidizations. If hagfishes and lampreys together form a monophyletic clade in the superclass Cyclostomata, as seems to be the case [55], this would mean that all vertebrates, including hagfish [56,57], share a common ancestor that had gone through 2R. To our knowledge, the opsin gene family is presently the only one that has been found to have retained a complete 2R quartet in a lamprey. Several previously investigated gene families in the literature display fewer family members in lampreys than in gnathostomes – see for instance [20,58], suggesting more extensive gene loss in the lamprey lineage after 2R. Nevertheless, some incomplete gene families are consistent with post-2R divergence of cyclostomes and jawed vertebrates, including the retinoic acid receptors RAR [54]. The genome-wide duplication pattern in the recently published sea lamprey (*Petromyzon marinus*) whole-genome sequence was found to be indicative of 2R [59].

## Conclusions

We present an extensive analysis of the paralogous chromosome regions housing the gene families for visual opsins, the G-protein alpha subunit families for transducin (GNAT) and adenylyl cyclase inhibition (GNAI), the oxytocin and vasopressin receptors (OT/VP-R), and the L-type voltage-gated calcium channel alpha subunits (CACNA1-L), using both phylogenetic analyses and positional data for these as well as 34 chromosomal neighboring gene families. This combined dataset makes it possible to make a robust inference how this region evolved. We conclude that these related chromosome regions originated from an ancestral chromosome that was duplicated in the two basal vertebrate tetraploidizations (2R) resulting in four paralogous chromosome regions. The paralogon was duplicated again in the teleost-specific tetraploidization (3R) giving teleost fish additional gene family members. We could detect extensive post-3R chromosomal rearrangements between the paralogous chromosome regions in teleost genomes that obscure the view of these whole genome duplications, as noted previously [45,46,53]. However, the analyses of extant teleost genomes combined with the data presented here from the spotted gar, an out-group to teleost evolution, allowed us to resolve the rearrangements.

Referring also to our previous analyses of several gene families, we conclude that the basal vertebrate tetraploidizations contributed with new genetic material in several gene families involved in the phototransduction cascade, but also in other functions related to the vertebrate nervous system.

## Methods

### Sequence identification and genome database searches

Amino acid sequence predictions of the visual opsin genes were retrieved from the Ensembl genome browser (http://www.ensembl.org) [60] from a representative selection of vertebrate clades: human (*Homo sapiens*), mouse (*Mus musculus*), grey short-tailed opossum (*Monodelphis domestica*), anole lizard (*Anolis carolinensis*), chicken (*Gallus gallus*), Western clawed frog (*Xenopus* (*Silurana*) *tropicalis*), coelacanth (*Latimeria chalumnae*), zebrafish (*Danio rerio*), three-spined stickleback (*Gasterosteus aculeatus*), medaka (*Oryzias latipes*) and spotted green pufferfish (*Tetraodon nigroviridis*). Amino acid sequence predictions from the spotted gar (*Lepisosteus oculatus*) genome were identified through BLAST searches against the LepOcu1 assembly available at the Pre! Ensembl genome browser (http://pre.ensembl.org/Lepisosteus_oculatus/Info/Index) and included in the analyses. Chicken data was complemented with cloned sequences (GenBank accession numbers: NP_990769.1, P22329.1 and P28682.1) [6,61]. Additionally, published visual opsin amino acid sequences from the pouched lamprey (*Geotria australis*) were used (GenBank accession numbers AAR14682.1, AAR14683.1, AAR14681.1, AAR14684.1 and AAR14680.1) [36].

For the oxytocin and vasopressin receptor gene family the same species repertoire as the visual opsins was used with a few exceptions: the Japanese pufferfish (*Takifugu rubripes*) was used instead of the related spotted green pufferfish and Southern platyfish (*Xiphophorus maculatus*) sequences (retrieved from Ensembl version 68) were added to increase resolution of the teleost branches. In addition, published oxytocin and vasopressin receptor sequences from the elephant shark (*Callorhinchus milii*) (GenBank accession numbers AB665985.1, AB665982.1, AB665983.1, AB665984.1, and AB671271.1) were added [29]. A sequence from the tunicate transparent sea squirt (*Ciona intestinalis*) was also added. Known OT/VP-R gene family members in the common octopus (*Octopus vulgaris*) were used as out-group, as described in [30].

For the CACNA1-L gene family the same repertoire as the visual opsins were used excluding Western clawed frog, coelacanth and spotted gar. Invertebrate sequences from transparent sea squirt and fruit fly (*Drosophila melanogaster*) were included for relative dating and as a root, respectively.

Sequences were identified in several Ensembl versions for the different gene families, using Ensembl’s automatic protein family predictions, spanning between Ensembl 60 (November 2010) to Ensembl 68 (July 2012). For the visual opsin gene family, gene predictions were identified through Ensembl’s gene tree function [62] searches since the *OPN* genes are spread among several Ensembl protein family predictions. All sequences and database identifiers have been verified against the genome assembly versions in Ensemble database version 71 (April 2013). This information can be found in Additional files 1 and 3. To identify additional family members that may have been excluded from the automatic Ensembl protein family predictions, Basic Local Alignment Searches (BLAST) [63] were performed using identified amino acid sequences (tblastn) as search terms with standard settings on the Ensembl database and the National Center for Biotechnology Information (NCBI) databases.

### Conserved synteny analysis

The locations of the identified visual opsin, OT/VP-R, GNAT, GNAI and CACNA1-L sequences were recorded for the genome assemblies detailed above. All locations were verified against the genome assembly versions in Ensemble database version 71 (April 2013). Using the BioMart function, lists of gene predictions corresponding to the chromosome blocks bearing these genes in the human genome assembly GRCh37 were downloaded from the Ensembl genome browser (versions specified below). This dataset was complemented with lists from the zebrafish genome (assembly Zv9) in some cases (see below). Chromosome blocks were defined as approximately 5 Mb in each direction of each of the genes in the above-mentioned gene families.

From these lists of syntenic gene predictions we identified Ensembl protein family predictions that had members on at least two of the included chromosome blocks: these protein family predictions represent the syntenic or neighboring gene families. Amino acid sequence predictions corresponding to each of the identified neighboring gene families were downloaded from the Ensembl genome browser in order to do amino acid sequence alignments and phylogenetic analyses, and the locations of all identified sequences were recorded. The species included in these analyses were: human, mouse, grey short-tailed opossum, chicken, three-spined stickleback, spotted green pufferfish, medaka, zebrafish, transparent sea squirt (*Ciona intestinalis* or *Ciona savignyi*) and fruit fly (*Drosophila melanogaster*). Sequence predictions from the following additional species were used in some families in order to ensure phylogenetic signal and/or taxonomic representation: Tasmanian devil (*Sarcophilus harrisii*), zebra finch (*Taeniopygia guttata*), anole lizard, Western clawed frog, Japanese pufferfish (*Takifugu rubripes*), Florida lancelet *(Branchiostoma floridae*) and the nematode *Caenorhabditis elegans*. Additional members that were not included in the automatic protein family predictions were identified by tblastn searches as described above. All sequences, database identifiers and locations have been verified against the genome assembly versions in Ensemble database version 71 (April 2013). For some of the neighboring gene families invertebrate sequences had to be identified by Hidden Markov Model searches (HMMER) using the HMMER web server (http://hmmer.janelia.org/) [64] and its pHMMER implementation against the UniProtKB database and the NCBI non-redundant (NR) protein database.

The identification of neighboring gene families was done independently for the regions corresponding to each of the visual opsin, OT/VP-R, GNAT/GNAI and CACNA1-L gene families. The neighboring gene families were subsequently pooled together into one dataset and used for the analyses of conserved synteny. Since several of the chromosome blocks used in the analyses overlapped, some of the gene families were identified twice.

### Selection of neighboring gene families in the visual opsin chromosome regions

The chromosome regions bearing the visual opsin genes *OPN1SW*, *RHO*, *OPN1LW* and *OPN1MW* in the human genome were used to select neighboring gene families. The chromosome blocks range between map positions 124 Mb and 134 Mb on chromosome 3, between map positions 123 Mb and 133 Mb on chromosome 7, and between map positions 148 Mb and 158 Mb on the X chromosome in Ensembl 60 (November 2010). Ensembl protein families with members present in at least two of the above-mentioned chromosomal regions were included in the subsequent phylogenetic analyses.

### Selection of neighboring gene families in the OT/VP-R chromosome regions

Since not all identified OT/VP-R genes could be identified in the human genome (Ocampo Daza et al 2012), both the zebrafish and the human genomes were used to identify neighboring gene families for the analysis of conserved synteny. Both sets of chromosome blocks were downloaded from Ensembl 60 (November 2010).

The chromosome blocks in the zebrafish genome range between map positions 3 Mb and 12.5 Mb as well as between map positions 12.5 Mb and 22 Mb on chromosome 4, between map positions 37 Mb and 52 Mb on chromosome 6, between map positions 13 Mb and 30 Mb on chromosome 23 and between map positions 1 bp and 7 Mb on chromosome 25. Since there are several OT/VP-R genes located on the same chromosomes, these blocks do not necessarily represent 5 Mb. For example*,* the *V1aR1-*type and *V2bR2*-type genes are both located on chromosome 4, but to investigate the paralogy relationship between these two genes the chromosome blocks were treated separately. Ensembl protein families with members on at least three of the chromosome blocks were selected for the analysis of conserved synteny.

In the human genome the chromosome blocks range between map positions 199 Mb and 209 Mb on chromosome 1, between map positions 3 Mb and 13 Mb on chromosome 3, between Map positions 56 Mb and 66 Mb on chromosome 12, and between map positions 147 Mb and 157 Mb on the X chromosome. Protein families with members on at least two of the chromosome blocks were selected.

### Selection of neighboring gene families in the CACNA1-L chromosome regions

A list of all gene predictions located in the chromosome blocks between map positions 196 Mb and 206 Mb on chromosome 1, 48.5 Mb and 58.5 Mb on chromosome 3, 1 bp and 7 Mb on chromosome 12 and between map positions 44 Mb and 54 Mb on chromosome X in the human genome were downloaded from Ensembl 61 (February 2011). These blocks represent the chromosomal regions of the *CACNA1S, CACNA1D, CACNA1C* and *CACNA1F* genes respectively. Ensembl protein families with members located on at least two of the chromosome blocks were considered for the analysis of conserved synteny.

### Selection of neighboring gene families in the GNAT and GNAI chromosome regions

Lists of genes in the *GNAT-GNAI* bearing chromosome regions were downloaded from Ensembl 59 (Aug 2010). The chromosome blocks range between map positions 74.8 Mb and 84.7 Mb on chromosome 7, between map positions 45.3 Mb and 55.2 Mb on chromosome 3, and between map positions 106 Mb and 115.1 Mb on chromosome 1 in the human genome. Ensembl protein families represented on at least two of the three human chromosome blocks were included in the analysis of conserved synteny.

### Sequence annotation and curation

For short, incomplete or highly diverging sequences among the identified gene predictions, the genomic sequences, including intronic and flanking sequences, were collected and the GenScan gene prediction server (http://genes.mit.edu/GENSCAN.html) [65] was used to ratify faulty exon predictions or to predict exons or entire genes *de novo*. Whenever possible, short Ensembl predictions were replaced with NCBI RefSeq sequences identified by BLAST searches, or with overlapping GenScan-predictions included in the Ensembl browser. Sequences that were still divergent with regard to exon-intron boundaries were curated manually by following consensus for splice donor and acceptor sites as well as sequence homology to other family members. Remaining highly divergent and unalignable sequence stretches in some of these predictions were removed. Short amino acid sequences that could not be ratified and did not provide enough sequence information in the alignments were removed entirely in order to prevent artifacts in the phylogenetic analyses. However the chromosomal locations of the gene predictions were registered.

### Sequence alignment and phylogenetic analyses

Amino acid sequences were aligned using the ClustalW algorithm (Gonnet weight matrix, gap opening penalty 10.0 and gap extension penalty 0.20) [66] or the MUSCLE algorithm (with 16 iterations) [67] and the resulting alignments were inspected manually in order to ratify faulty or divergent predictions and curate misaligned sequence stretches. The manually curated alignments were used to calculate phylogenetic trees, using both the Neighbor Joining (NJ) and Phylogenetic Maximum Likelihood (PhyML) methods.

NJ trees with non-parametric bootstrap support were made using standard settings (NJ clustering algorithm with 1000 bootstrap iterations) in ClustalX 2.0.12 [66]. PhyML trees were made using the PhyML3.0 algorithm [68] with the following settings: amino acid frequencies (equilibrium frequencies), proportion of invariable sites (with optimized *p-invar*) and gamma shape parameters were estimated from the alignments, the number of substitution rate categories was set to 8, BIONJ was chosen to create the starting tree, both NNI and SPR tree optimization methods were considered and both tree topology and branch length optimization were chosen. The amino acid substitution model was selected for each alignment using ProtTest3.2 [69] with the following settings: Likelihood scores were computed selecting between the JTT, LG, DCMut, Dayhoff, WAG, Blosum62 and VT substitution model matrices with no add-ons and a Fixed BioNJ JTT-based starting tree. Based on these analyses the LG model was chosen for the visual opsin gene family and the JTT model for the OT/VP-R and CACNA1-L gene families. The JTT model was also chosen for the majority of the neighboring gene families, except for the B4GALNT, CACNA2D, COL, L1CAM, PLG, PPP, QSOX and UBA gene families where the WAG model was chosen, and the RPL and TWF gene families where the LG model was chosen. The visual opsin, OT/VP-R and CACNA1-L PhyML topologies are supported by non-parametric bootstrap tests with 100 iterations. The PhyML tree topologies for the neighboring families are supported by non-parametric SH-like approximate likelihood ratio (aLRT) tests [68,70] since this method is faster.

### Relative dating and outgroup choice

For the majority of the phylogenetic analyses the identified fruit fly sequences were used as out-group to root the trees. In some cases where no fruit fly sequence could be identified, *C. elegans* or tunicate sequences were used instead. The inclusion of lancelet or tunicate sequences in the phylogenetic analyses provides the relative dating for the time-window of 2R. For a few families where no invertebrate sequences could be identified, midpoint-rooting [71] was used. The rooting of the phylogenetic trees for the neighboring gene families is summarized in Table 2. The OT/VP-R gene family was rooted with identified *Octopus vulgaris* family members, as described in [30]. The visual opsin gene family was rooted with the human *OPN3* gene.

### Analysis of conserved synteny between the human and spotted gar genomes

A FASTA file containing all preliminary protein sequence predictions from the spotted gar genome assembly (LepOcu1) available in the Pre! Ensembl database was downloaded using the FTP server (*http://pre.ensembl.org/downloads.html*, retrieved July 25, 2012). This file was used for orthology searches using InParanoid 4.1 [72,73] (with standard settings) against a FASTA file containing translations of all human canonical transcripts (available from the InParanoid website: *http://inparanoid.sbc.su.se/download/7.0_current/sequences/processed/H.sapiens.fa*. From the resulting global dataset of orthology matches between the spotted gar and human genomes, the best spotted gar matches (highest bootstrap support) for each human gene prediction used in this study were mined. The locations of each of these spotted gar protein prediction matches in the spotted gar genome assembly were recorded and charted.

### Description of additional files

The data sets supporting the results of this article, including all sequence alignments and phylogenetic tree files, are available in figshare and have been cited in the article where appropriate – see references [35,42-44]. The following additional files are included with this article. Additional file 1 includes detailed information on the main gene families, such as database identifiers, location data and annotation notes for all analyzed sequences. Additional file 2 includes the phylogenetic trees of the main gene families (Figures S1-S8). Additional file 3 includes detailed information on the neighboring gene families. Additional file 4 includes supplementary notes on the phylogenies of neighboring gene families. Additional file 5 includes conserved synteny tables with the synteny data underlying our evolutionary scenario.

## Competing interests

The authors declare that they have no competing interests.

## Authors’ contributions

DLag, DOD, JW and GS participated in the study design and performed phylogenetic and chromosome analyses. XMA participated in the analyses and in the discussion of results. DLar conceived and co-designed the study and participated in the analyses. All authors co-wrote the article and have read and approved the final version.

## Supplementary Material

Additional file 1 **Tables including detailed information on the main gene families.** Includes database identifiers, location data and annotation notes for all analyzed sequences. Click here for file

Additional file 2 **Figures S1-S8.** Phylogenetic trees of the main gene families of visual opsins, oxytocin and vasopressin receptors (OT/VP-R) and L-type voltage-gated calcium channels (CACNA1-L). Full lists of sequence names, locations and database identifiers for these families are included in Additional file 1. Click here for file

Additional file 3 **Tables including detailed information on the neighboring gene families.** Includes database identifiers, location data and annotation notes for all analyzed sequences. Click here for file

Additional file 4 **Supplementary notes.** Topologies of neighboring gene family trees. Click here for file

Additional file 5 **Tables of conserved synteny between the identified chromosome blocks in the human, chicken, spotted gar, zebrafish and three-spined stickleback genomes.** Each table is included as a separate tab in the workbook. Click here for file
