# Supplementary material for: The vertebrate ancestral repertoire of visual opsins, transducin alpha subunits and oxytocin/vasopressin receptors was established by duplication of their shared genomic region in the two rounds of early vertebrate genome duplications
Source: BMC Evol Biol. 2013 Nov 2;13:238. doi: 10.1186/1471-2148-13-238 (PMC3826523; doi:10.1186/1471-2148-13-238)
Supplement: Additional file 2 — Figures S1-S8. Phylogenetic trees of the main gene families of visual opsins, oxytocin and vasopressin receptors (OT/VP-R) and L-type voltage-gated calcium channels (CACNA1-L). Full lists of sequence names, locations and database identifiers for these families are included in Additional file 1. [file 1471-2148-13-238-S2.pdf]

FIGURES S1-S8

Figure S1: Neighbor joining tree of visual opsin genes of the *LWS, SWS1, SWS2, RH1* and *RH2* clades. The tree is supported by a non-parametric bootstrap analysis with 100 replicates. Bootstrap values shown at the nodes. The tree was rooted with the human *OPN3* sequence. For the sequence names, species abbreviations are applied as described in *Methods*, followed by the number of the chromosome or linkage group where the gene is located (if known) and the gene/subtype name (see Table 1). Scale bar indicate phylogenetic distance as number of substitutions per site. The phylogenetic tree file and the alignment it was made from are available from: http://dx.doi.org/10.6084/m9.figshare.705157.

Figure S2: Phylogenetic Maximum Likelihood tree of visual opsin genes of the *LWS, SWS1, SWS2, RH1* and *RH2* clades. The tree is supported by a non-parametric bootstrap analysis with 100 replicates. Bootstrap values shown at the nodes. The tree was rooted with the human *OPN3* sequence. For the sequence names, species abbreviations are applied as described in *Methods*, followed by the number of the chromosome or linkage group where the gene is located (if known) and the gene/subtype name (see Table 1). Scale bar indicate phylogenetic distance as number of substitutions per site. The phylogenetic tree file and the alignment it was made from are available from: http://dx.doi.org/10.6084/m9.figshare.705157.

Figure S3: Neighbor joining tree of visual opsin genes of the *LWS, SWS1, SWS2, RH1* and *RH2* clades as well as vertebrate ancient (V/A) opsins, pinopsins and *Ciona intestinalis* opsins (Ci-opsins). The tree is supported by a non-parametric bootstrap analysis with 1000 replicates. Bootstrap values shown at the nodes. The tree was rooted with the human *OPN3* sequence. Sequence names as in Figures S1 and S2. See Supplemental data 1 for a full list of sequence names and locations, including V/A opsins, pinopsins and Ci-opsins. The V/A opsins form a basal clade that clusters with the Ci-opsins. The pinopsins form a well-supported clade that clusters with the *LWS* opsins, although with weak support. Scale bar indicate phylogenetic distance as number of substitutions per site. The phylogenetic tree file and the alignment it was made from are available from: http://dx.doi.org/10.6084/m9.figshare.705157.

Figure S4: Phylogenetic Maximum Likelihood tree of visual opsin genes of the *LWS, SWS1, SWS2, RH1* and *RH2* clades as well as vertebrate ancient (V/A) opsins, pinopsins and *Ciona intestinalis* opsins (Ci-opsins). The tree is supported by a non-parametric bootstrap analysis with 100 replicates. Bootstrap values shown at the nodes. The tree was rooted with the human *OPN3* sequence. Sequence names as in Figures S1 and S2. See Supplemental data 1 for a full list of sequence names and locations, including V/A opsins, pinopsins and Ci-opsins. The V/A opsins, pinopsins and Ci-opsins form well-supported clades that cluster with the *LWS* opsins. As in the NJ analysis (Fig. S3) the Ci-opsins cluster basal to the V/A opsin clade. Scale bar indicate phylogenetic distance as number of substitutions per site. The phylogenetic tree file and the alignment it was made from are available from: http://dx.doi.org/10.6084/m9.figshare.705157.

Figure S5: Neighbor joining tree of oxytocin and vasopressin receptor (OT/VP-R) subtype genes. The tree is supported by a non-parametric bootstrap analysis with 1000 replicates. Bootstrap values shown at the nodes. Red values indicate nodes with bootstrap values lower than 50% that were not considered informative. The tree was rooted with the octopus *OTR, CPR1* and *CPR2* sequences. The V2C sequences do not form a well-supported clade; this is indicated by blue branches. Scale bar indicate phylogenetic distance as number of substitutions per site. The phylogenetic tree file and the alignment it was made from are available from: http://dx.doi.org/10.6084/m9.figshare.707336.

Figure S6: Phylogenetic Maximum Likelihood tree of oxytocin and vasopressin receptor (OT/VP-R) subtype genes. The tree is supported by a non-parametric bootstrap analysis with 100 replicates. Bootstrap values shown at the nodes. Red values indicate nodes with bootstrap values lower than 50% that were not considered informative. The tree was rooted with the octopus *OTR, CPR1* and *CPR2* sequences. The V2C sequences do not form a well-supported clade; this is indicated by blue branches. Scale bar indicate phylogenetic distance as number of substitutions per site. The phylogenetic tree file and the alignment it was made from are available from: http://dx.doi.org/10.6084/m9.figshare.707336.

Figure S7. Neighbor joining tree of voltage-gated calcium channel L-type alpha 1 subunit (CACNA1-L) genes. The tree is supported by a non-parametric bootstrap analysis with 1000 replicates. Bootstrap values shown at the nodes. All nodes are supported by over 50% of bootstrap iterations. The tree was rooted with the fruit fly *Ca-α1D* sequence. Scale bar indicate phylogenetic distance as number of substitutions per site. The phylogenetic tree file and the alignment it was made from are available from: http://dx.doi.org/10.6084/m9.figshare.710637.

Figure S8. Phylogenetic Maximum Likelihood tree of voltage-gated calcium channel L-type alpha 1 subunit (CACNA1-L) genes. The tree is supported by a non-parametric bootstrap analysis with 100 replicates. Bootstrap values shown at the nodes. All nodes are supported by over 50% of bootstrap iterations. The tree was rooted with the fruit fly *Ca-α1D* sequence. In this tree the CACNA1D sequences do not form one well-supported clade; this is indicated by blue branches. Scale bar indicate phylogenetic distance as number of substitutions per site. The phylogenetic tree file and the alignment it was made from are available from: http://dx.doi.org/10.6084/m9.figshare.710637.

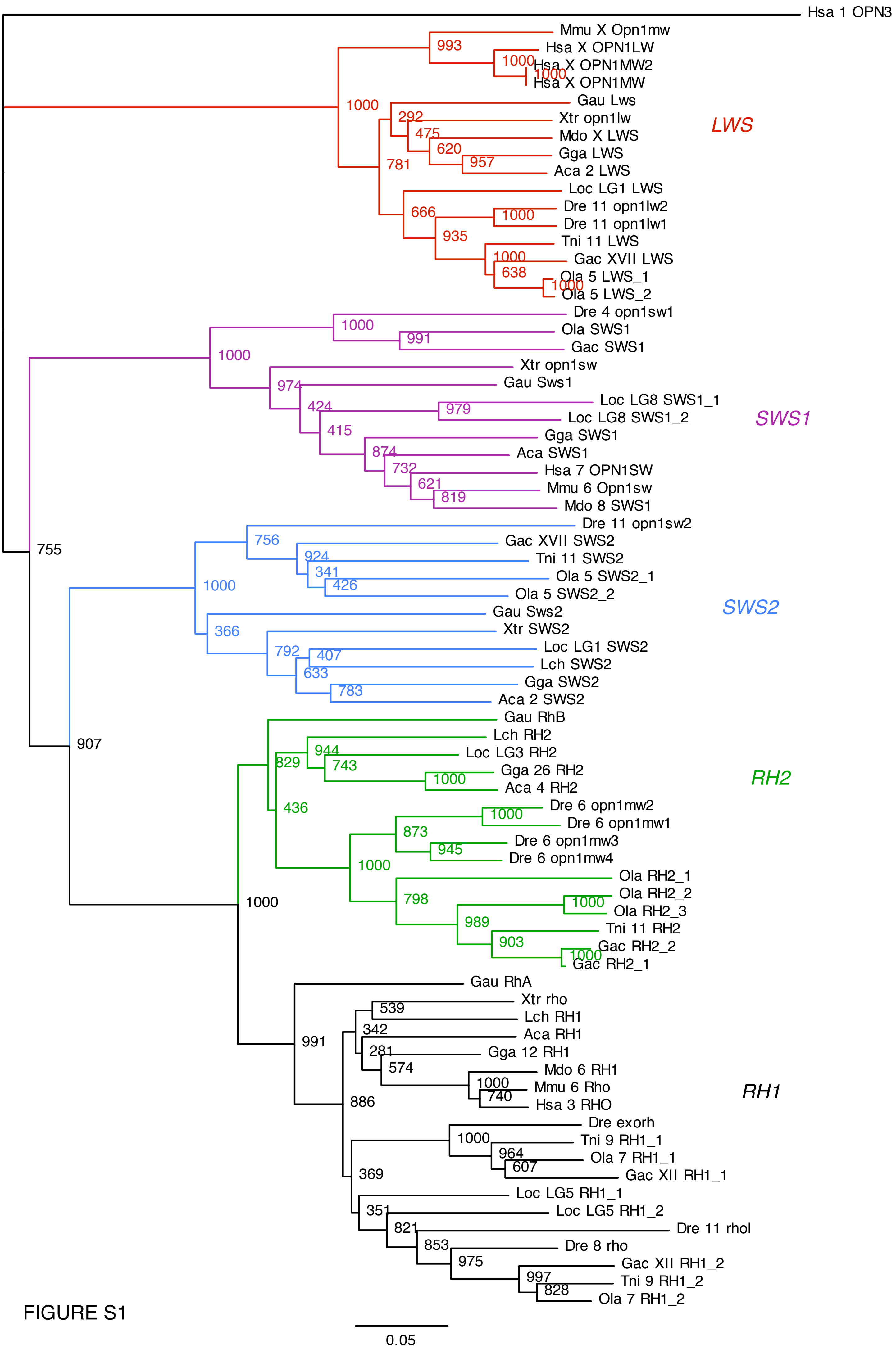
Hsa 1 OPN3
Mmu X Opn1mw
Hsa X OPN1LW
Hsa X OPN1MW2
Hsa X OPN1MW
Gau Lws
Xtr opn1lw
Mdo X LWS
Gga LWS
Aca 2 LWS
Loc LG1 LWS
Dre 11 opn1lw2
Dre 11 opn1lw1
Tni 11 LWS
Gac XVII LWS
Ola 5 LWS_1
Ola 5 LWS_2
LWS
Dre 4 opn1sw1
Ola SWS1
Gac SWS1
Xtr opn1sw
Gau Sws1
Loc LG8 SWS1_1
Loc LG8 SWS1_2
Gga SWS1
Aca SWS1
Hsa 7 OPN1SW
Mmu 6 Opn1sw
Mdo 8 SWS1
SWS1
Dre 11 opn1sw2
Gac XVII SWS2
Tni 11 SWS2
Ola 5 SWS2_1
Ola 5 SWS2_2
Gau Sws2
Xtr SWS2
Loc LG1 SWS2
Lch SWS2
Gga SWS2
Aca 2 SWS2
SWS2
Gau RhB
Lch RH2
Loc LG3 RH2
Gga 26 RH2
Aca 4 RH2
Dre 6 opn1mw2
Dre 6 opn1mw1
Dre 6 opn1mw3
Dre 6 opn1mw4
Ola RH2_1
Ola RH2_2
Ola RH2_3
Tni 11 RH2
Gac RH2_2
Gac RH2_1
RH2
Gau RhA
Xtr rho
Lch RH1
Aca RH1
Gga 12 RH1
Mdo 6 RH1
Mmu 6 Rho
Hsa 3 RHO
RH1
Dre exorh
Tni 9 RH1_1
Ola 7 RH1_1
Gac XII RH1_1
Loc LG5 RH1_1
Loc LG5 RH1_2
Dre 11 rhol
Dre 8 rho
Gac XII RH1_2
Tni 9 RH1_2
Ola 7 RH1_2
0.05

FIGURE S1

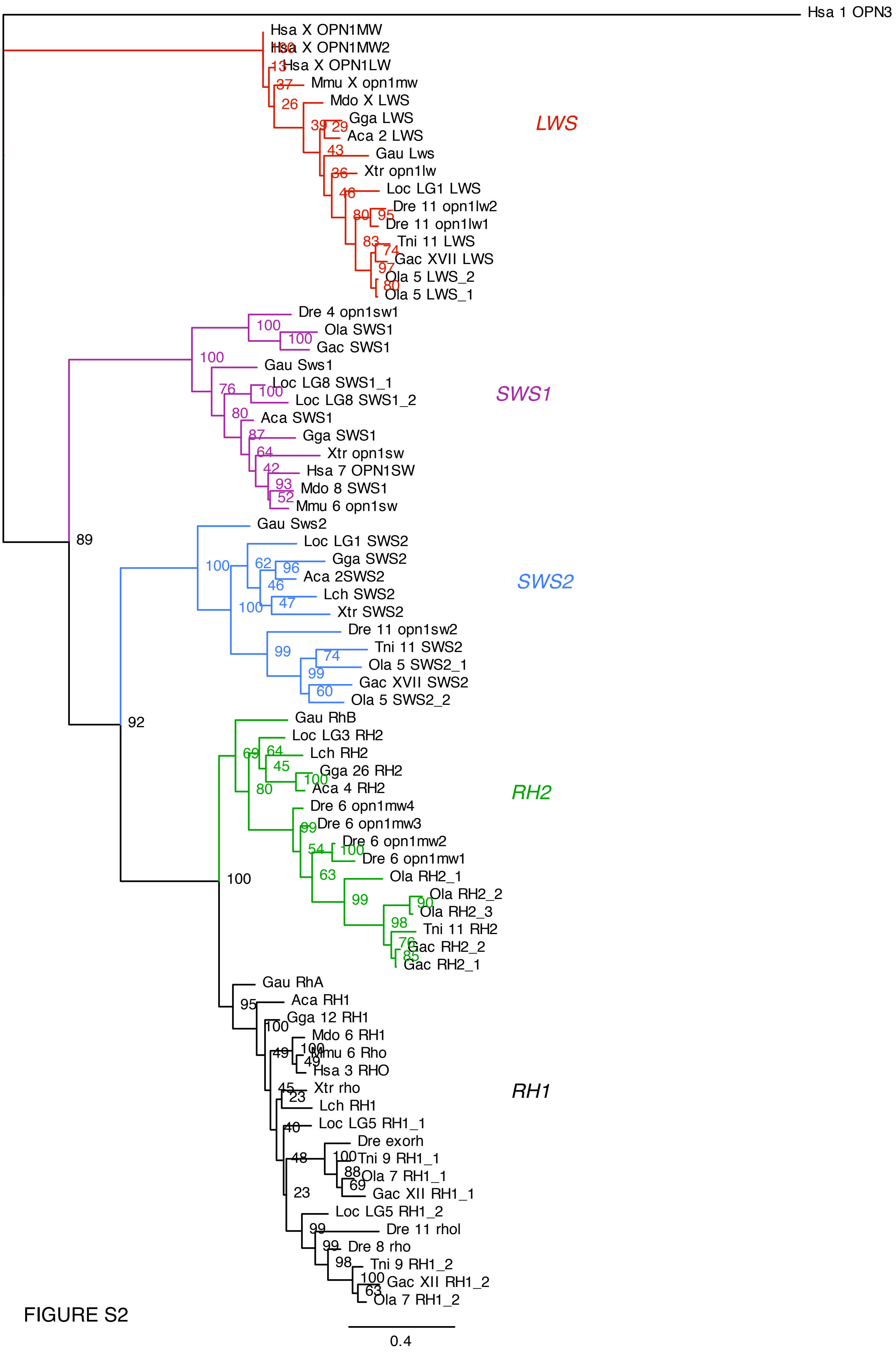

Hsa 1 OPN3
Hsa X OPN1MW
Hsa X OPN1MW2
Hsa X OPN1LW
Mmu X opn1mw
Mdo X LWS
Gga LWS
Aca 2 LWS
Gau Lws
Xtr opn1lw
Loc LG1 LWS
Dre 11 opn1lw2
Dre 11 opn1lw1
Tni 11 LWS
Gac XVII LWS
Ola 5 LWS_2
Ola 5 LWS_1
LWS
Dre 4 opn1sw1
Ola SWS1
Gac SWS1
Gau Sws1
Loc LG8 SWS1_1
Loc LG8 SWS1_2
Aca SWS1
Gga SWS1
Xtr opn1sw
Hsa 7 OPN1SW
Mdo 8 SWS1
Mmu 6 opn1sw
SWS1
Gau Sws2
Loc LG1 SWS2
Gga SWS2
Aca 2SWS2
Lch SWS2
Xtr SWS2
Dre 11 opn1sw2
Tni 11 SWS2
Ola 5 SWS2_1
Gac XVII SWS2
Ola 5 SWS2_2
SWS2
Gau RhB
Loc LG3 RH2
Lch RH2
Gga 26 RH2
Aca 4 RH2
Dre 6 opn1mw4
Dre 6 opn1mw3
Dre 6 opn1mw2
Dre 6 opn1mw1
Ola RH2_1
Ola RH2_2
Ola RH2_3
Tni 11 RH2
Gac RH2_2
Gac RH2_1
RH2
Gau RhA
Aca RH1
Gga 12 RH1
Mdo 6 RH1
Mmu 6 Rho
Hsa 3 RHO
Xtr rho
Lch RH1
Loc LG5 RH1_1
Dre exorh
Tni 9 RH1_1
Ola 7 RH1_1
Gac XII RH1_1
Loc LG5 RH1_2
Dre 11 rhol
Dre 8 rho
Tni 9 RH1_2
Gac XII RH1_2
Ola 7 RH1_2
RH1
89
92
100
0.4

FIGURE S2

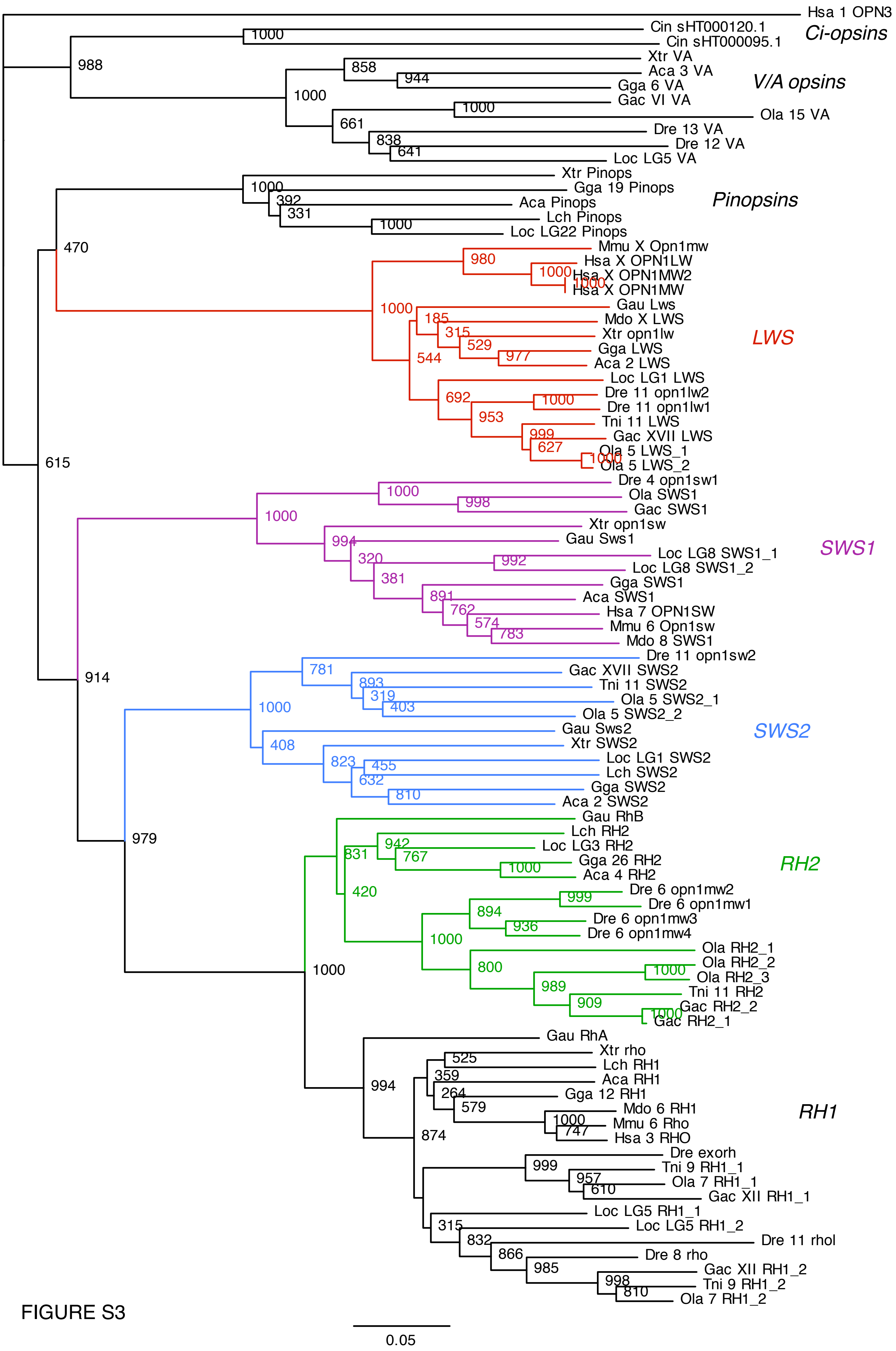
Hsa 1 OPN3
Cin sHT000120.1
Cin sHT000095.1
Ci-opsins
Xtr VA
Aca 3 VA
Gga 6 VA
Gac VI VA
V/A opsins
Ola 15 VA
Dre 13 VA
Dre 12 VA
Loc LG5 VA
Xtr Pinops
Gga 19 Pinops
Aca Pinops
Pinopsins
Lch Pinops
Loc LG22 Pinops
Mmu X Opn1mw
Hsa X OPN1LW
Hsa X OPN1MW2
Hsa X OPN1MW
Gau Lws
Mdo X LWS
Xtr opn1lw
Gga LWS
Aca 2 LWS
LWS
Loc LG1 LWS
Dre 11 opn1lw2
Dre 11 opn1lw1
Tni 11 LWS
Gac XVII LWS
Ola 5 LWS_1
Ola 5 LWS_2
Dre 4 opn1sw1
Ola SWS1
Gac SWS1
Xtr opn1sw
Gau Sws1
Loc LG8 SWS1_1
Loc LG8 SWS1_2
SWS1
Gga SWS1
Aca SWS1
Hsa 7 OPN1SW
Mmu 6 Opn1sw
Mdo 8 SWS1
Dre 11 opn1sw2
Gac XVII SWS2
Tni 11 SWS2
Ola 5 SWS2_1
Ola 5 SWS2_2
Gau Sws2
Xtr SWS2
SWS2
Loc LG1 SWS2
Lch SWS2
Gga SWS2
Aca 2 SWS2
Gau RhB
Lch RH2
Loc LG3 RH2
Gga 26 RH2
Aca 4 RH2
RH2
Dre 6 opn1mw2
Dre 6 opn1mw1
Dre 6 opn1mw3
Dre 6 opn1mw4
Ola RH2_1
Ola RH2_2
Ola RH2_3
Tni 11 RH2
Gac RH2_2
Gac RH2_1
Gau RhA
Xtr rho
Lch RH1
Aca RH1
Gga 12 RH1
Mdo 6 RH1
Mmu 6 Rho
Hsa 3 RHO
RH1
Dre exorh
Tni 9 RH1_1
Ola 7 RH1_1
Gac XII RH1_1
Loc LG5 RH1_1
Loc LG5 RH1_2
Dre 11 rhol
Dre 8 rho
Gac XII RH1_2
Tni 9 RH1_2
Ola 7 RH1_2
0.05

FIGURE S3

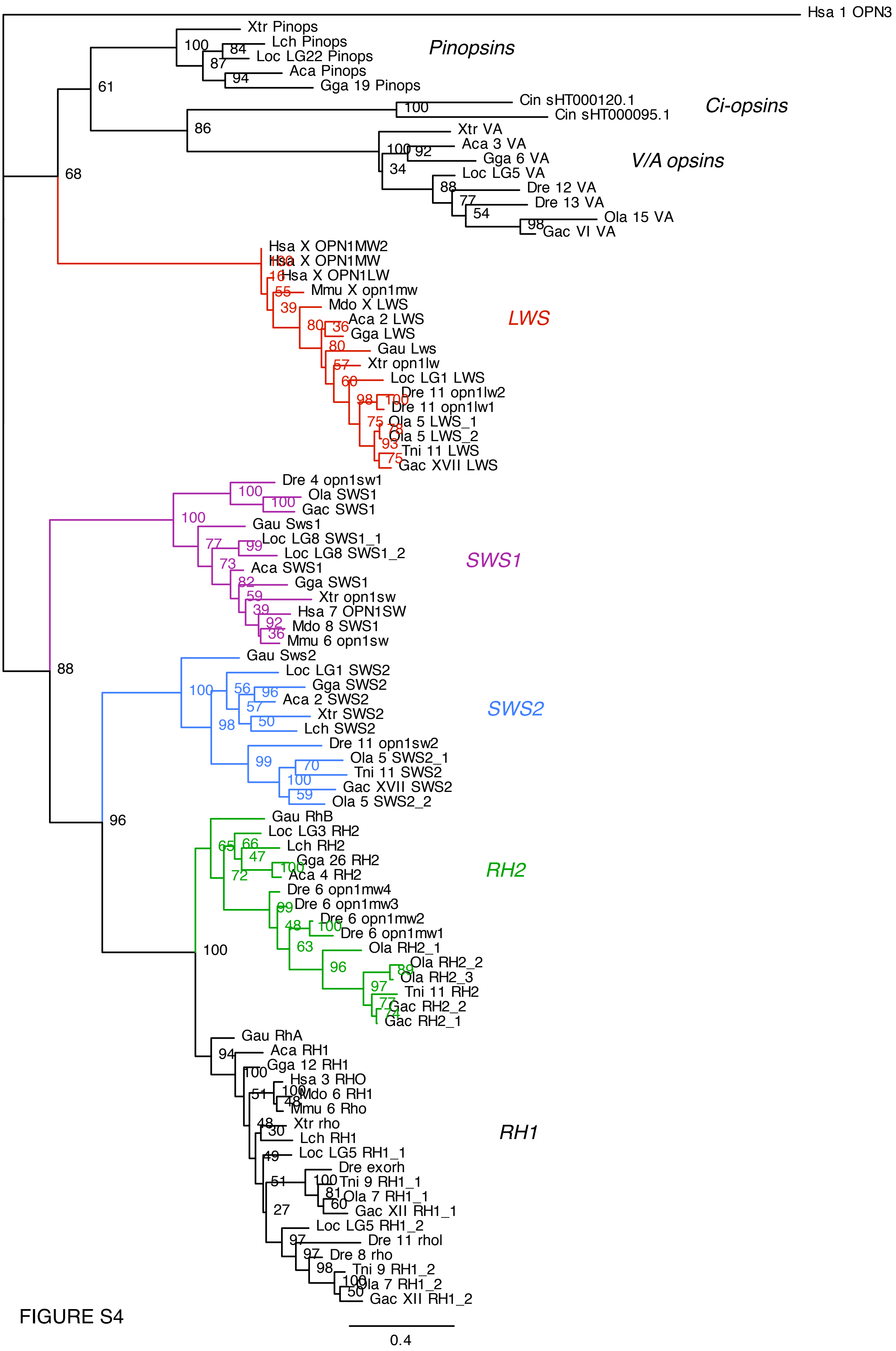

Hsa 1 OPN3
Xtr Pinops
Lch Pinops
Loc LG22 Pinops
Aca Pinops
Gga 19 Pinops
Pinopsins
Cin sHT000120.1
Cin sHT000095.1
Ci-opsins
Xtr VA
Aca 3 VA
Gga 6 VA
Loc LG5 VA
Dre 12 VA
Dre 13 VA
Ola 15 VA
Gac VI VA
V/A opsins
Hsa X OPN1MW2
Hsa X OPN1MW
Hsa X OPN1LW
Mmu X opn1mw
Mdo X LWS
Aca 2 LWS
Gga LWS
Gau Lws
Xtr opn1lw
Loc LG1 LWS
Dre 11 opn1lw2
Dre 11 opn1lw1
Ola 5 LWS_1
Ola 5 LWS_2
Tni 11 LWS
Gac XVII LWS
LWS
Dre 4 opn1sw1
Ola SWS1
Gac SWS1
Gau Sws1
Loc LG8 SWS1_1
Loc LG8 SWS1_2
Aca SWS1
Gga SWS1
Xtr opn1sw
Hsa 7 OPN1SW
Mdo 8 SWS1
Mmu 6 opn1sw
SWS1
Gau Sws2
Loc LG1 SWS2
Gga SWS2
Aca 2 SWS2
Xtr SWS2
Lch SWS2
Dre 11 opn1sw2
Ola 5 SWS2_1
Tni 11 SWS2
Gac XVII SWS2
Ola 5 SWS2_2
SWS2
Gau RhB
Loc LG3 RH2
Lch RH2
Gga 26 RH2
Aca 4 RH2
Dre 6 opn1mw4
Dre 6 opn1mw3
Dre 6 opn1mw2
Dre 6 opn1mw1
Ola RH2_1
Ola RH2_2
Ola RH2_3
Tni 11 RH2
Gac RH2_2
Gac RH2_1
RH2
Gau RhA
Aca RH1
Gga 12 RH1
Hsa 3 RHO
Mdo 6 RH1
Mmu 6 Rho
Xtr rho
Lch RH1
Loc LG5 RH1_1
Dre exorh
Tni 9 RH1_1
Ola 7 RH1_1
Gac XII RH1_1
Loc LG5 RH1_2
Dre 11 rhol
Dre 8 rho
Tni 9 RH1_2
Ola 7 RH1_2
Gac XII RH1_2
RH1
0.4

FIGURE S4

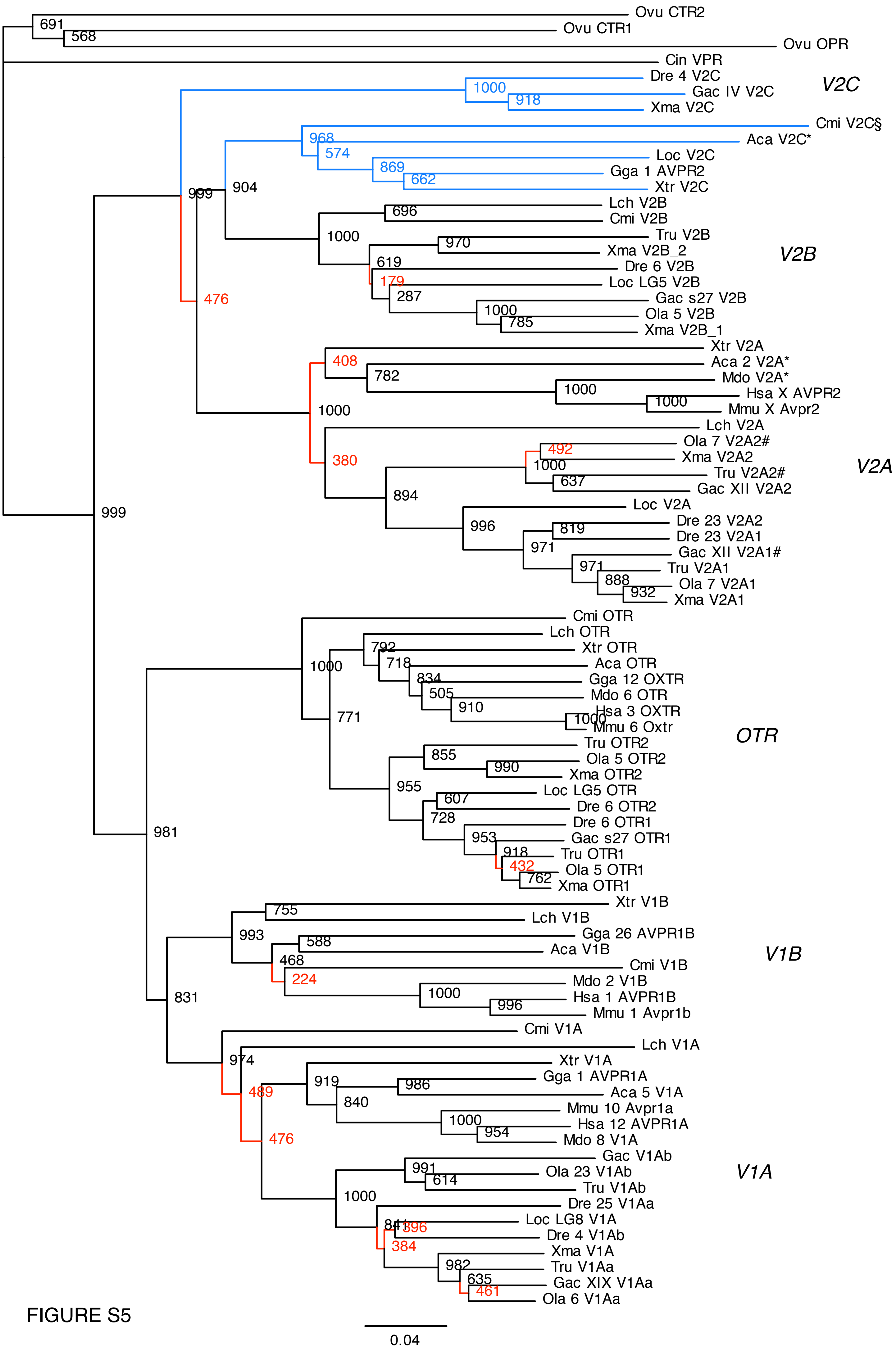
Ovu CTR2
Ovu CTR1
Ovu OPR
Cin VPR
Dre 4 V2C
Gac IV V2C
Xma V2C
V2C
Cmi V2C§
Aca V2C*
Loc V2C
Gga 1 AVPR2
Xtr V2C
Lch V2B
Cmi V2B
Tru V2B
Xma V2B_2
Dre 6 V2B
Loc LG5 V2B
Gac s27 V2B
Ola 5 V2B
Xma V2B_1
V2B
Xtr V2A
Aca 2 V2A*
Mdo V2A*
Hsa X AVPR2
Mmu X Avpr2
Lch V2A
Ola 7 V2A2#
Xma V2A2
Tru V2A2#
Gac XII V2A2
Loc V2A
Dre 23 V2A2
Dre 23 V2A1
Gac XII V2A1#
Tru V2A1
Ola 7 V2A1
Xma V2A1
V2A
Cmi OTR
Lch OTR
Xtr OTR
Aca OTR
Gga 12 OXTR
Mdo 6 OTR
Hsa 3 OXTR
Mmu 6 Oxtr
Tru OTR2
Ola 5 OTR2
Xma OTR2
Loc LG5 OTR
Dre 6 OTR2
Dre 6 OTR1
Gac s27 OTR1
Tru OTR1
Ola 5 OTR1
Xma OTR1
OTR
Xtr V1B
Lch V1B
Gga 26 AVPR1B
Aca V1B
Cmi V1B
Mdo 2 V1B
Hsa 1 AVPR1B
Mmu 1 Avpr1b
V1B
Cmi V1A
Lch V1A
Xtr V1A
Gga 1 AVPR1A
Aca 5 V1A
Mmu 10 Avpr1a
Hsa 12 AVPR1A
Mdo 8 V1A
Gac V1Ab
Ola 23 V1Ab
Tru V1Ab
Dre 25 V1Aa
Loc LG8 V1A
Dre 4 V1Ab
Xma V1A
Tru V1Aa
Gac XIX V1Aa
Ola 6 V1Aa
V1A
691
568
1000
918
968
574
869
662
999
904
476
696
1000
970
619
179
287
1000
785
408
782
1000
1000
1000
380
492
1000
637
894
996
819
971
971
888
932
999
1000
792
718
834
505
910
1000
771
855
990
955
607
728
953
918
432
762
981
755
993
588
468
224
1000
996
831
974
489
919
986
840
1000
954
476
991
614
1000
841
396
384
982
635
461
0.04

FIGURE S5

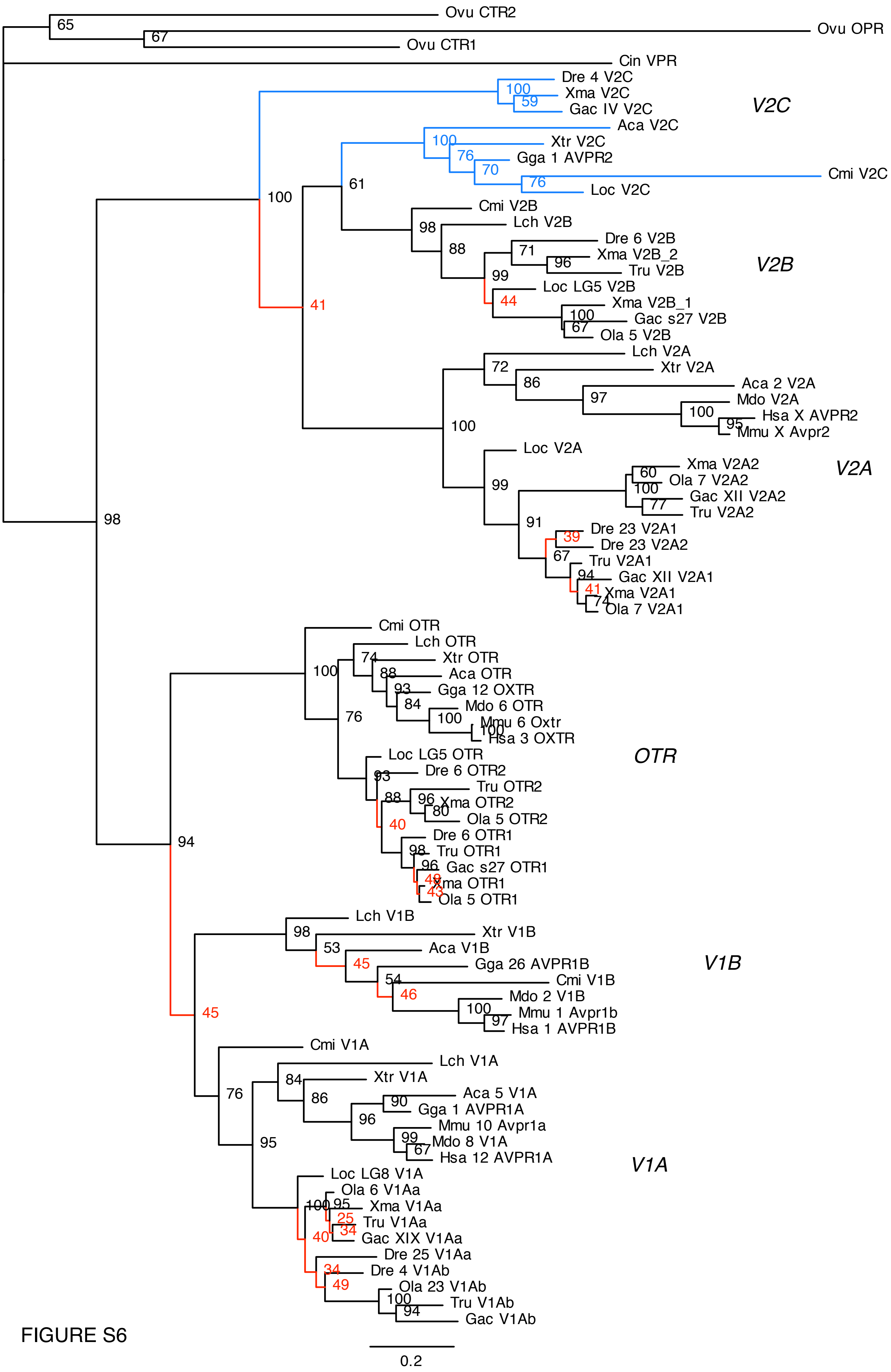

Ovu CTR2
Ovu OPR
Ovu CTR1
Cin VPR
Dre 4 V2C
Xma V2C
Gac IV V2C
V2C
Aca V2C
Xtr V2C
Gga 1 AVPR2
Cmi V2C
Loc V2C
Cmi V2B
Lch V2B
Dre 6 V2B
Xma V2B_2
Tru V2B
V2B
Loc LG5 V2B
Xma V2B_1
Gac s27 V2B
Ola 5 V2B
Lch V2A
Xtr V2A
Aca 2 V2A
Mdo V2A
Hsa X AVPR2
Mmu X Avpr2
Loc V2A
Xma V2A2
V2A
Ola 7 V2A2
Gac XII V2A2
Tru V2A2
Dre 23 V2A1
Dre 23 V2A2
Tru V2A1
Gac XII V2A1
Xma V2A1
Ola 7 V2A1
Cmi OTR
Lch OTR
Xtr OTR
Aca OTR
Gga 12 OXTR
Mdo 6 OTR
Mmu 6 Oxtr
Hsa 3 OXTR
Loc LG5 OTR
OTR
Dre 6 OTR2
Tru OTR2
Xma OTR2
Ola 5 OTR2
Dre 6 OTR1
Tru OTR1
Gac s27 OTR1
Xma OTR1
Ola 5 OTR1
Lch V1B
Xtr V1B
Aca V1B
Gga 26 AVPR1B
V1B
Cmi V1B
Mdo 2 V1B
Mmu 1 Avpr1b
Hsa 1 AVPR1B
Cmi V1A
Lch V1A
Xtr V1A
Aca 5 V1A
Gga 1 AVPR1A
Mmu 10 Avpr1a
Mdo 8 V1A
Hsa 12 AVPR1A
V1A
Loc LG8 V1A
Ola 6 V1Aa
Xma V1Aa
Tru V1Aa
Gac XIX V1Aa
Dre 25 V1Aa
Dre 4 V1Ab
Ola 23 V1Ab
Tru V1Ab
Gac V1Ab
0.2

FIGURE S6

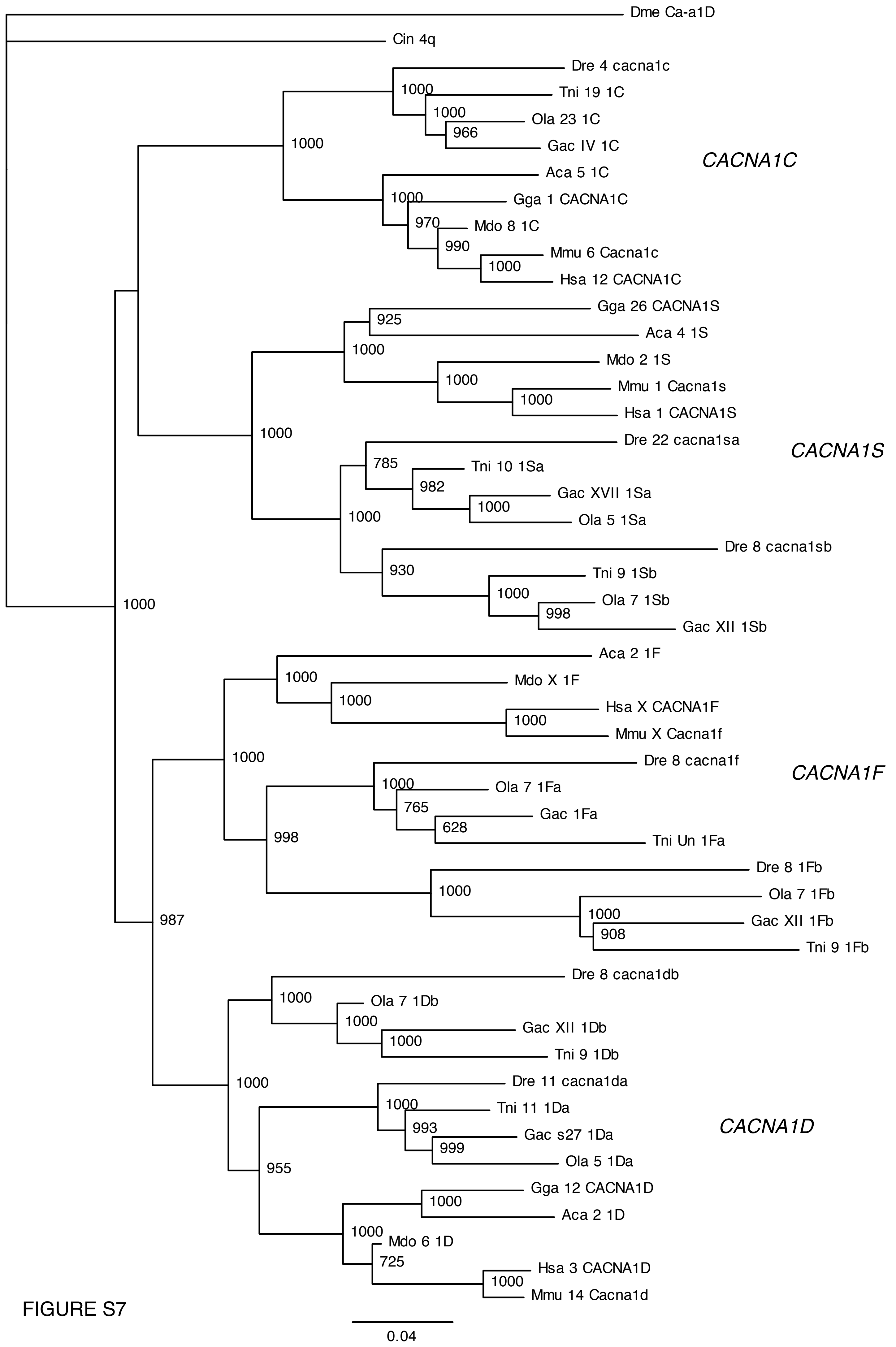

Dme Ca-a1D
Cin 4q
Dre 4 cacna1c
Tni 19 1C
Ola 23 1C
Gac IV 1C
Aca 5 1C
Gga 1 CACNA1C
Mdo 8 1C
Mmu 6 Cacna1c
Hsa 12 CACNA1C
CACNA1C
Gga 26 CACNA1S
Aca 4 1S
Mdo 2 1S
Mmu 1 Cacna1s
Hsa 1 CACNA1S
Dre 22 cacna1sa
Tni 10 1Sa
Gac XVII 1Sa
Ola 5 1Sa
CACNA1S
Dre 8 cacna1sb
Tni 9 1Sb
Ola 7 1Sb
Gac XII 1Sb
Aca 2 1F
Mdo X 1F
Hsa X CACNA1F
Mmu X Cacna1f
Dre 8 cacna1f
Ola 7 1Fa
Gac 1Fa
Tni Un 1Fa
CACNA1F
Dre 8 1Fb
Ola 7 1Fb
Gac XII 1Fb
Tni 9 1Fb
Dre 8 cacna1db
Ola 7 1Db
Gac XII 1Db
Tni 9 1Db
Dre 11 cacna1da
Tni 11 1Da
Gac s27 1Da
Ola 5 1Da
CACNA1D
Gga 12 CACNA1D
Aca 2 1D
Mdo 6 1D
Hsa 3 CACNA1D
Mmu 14 Cacna1d
1000
966
970
990
925
785
982
930
998
987
998
765
628
1000
955
993
999
725
0.04

FIGURE S7

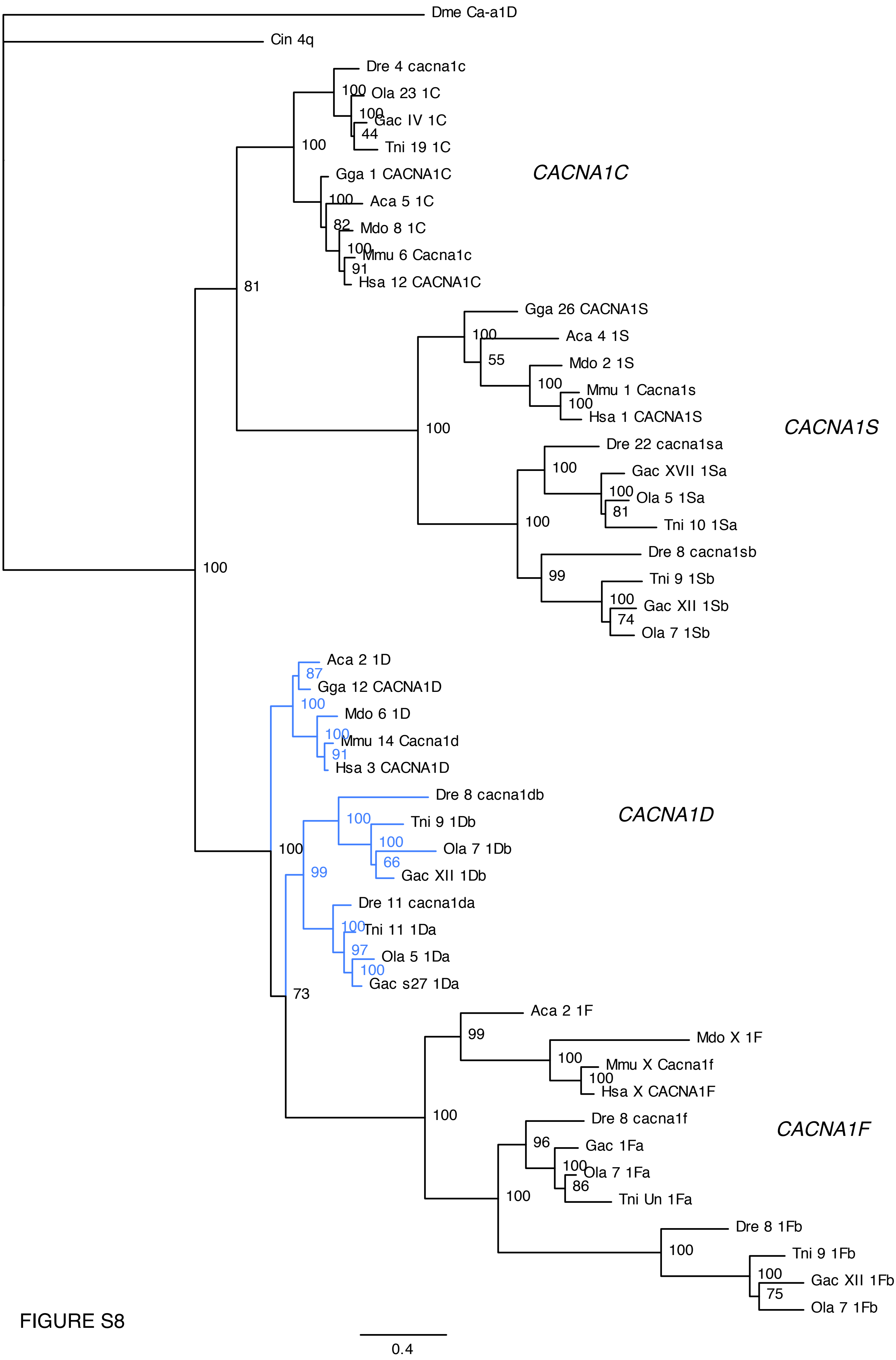

Dme Ca-a1D
Cin 4q
Dre 4 cacna1c
Ola 23 1C
Gac IV 1C
Tni 19 1C
Gga 1 CACNA1C
Aca 5 1C
Mdo 8 1C
Mmu 6 Cacna1c
Hsa 12 CACNA1C
CACNA1C
Gga 26 CACNA1S
Aca 4 1S
Mdo 2 1S
Mmu 1 Cacna1s
Hsa 1 CACNA1S
CACNA1S
Dre 22 cacna1sa
Gac XVII 1Sa
Ola 5 1Sa
Tni 10 1Sa
Dre 8 cacna1sb
Tni 9 1Sb
Gac XII 1Sb
Ola 7 1Sb
Aca 2 1D
Gga 12 CACNA1D
Mdo 6 1D
Mmu 14 Cacna1d
Hsa 3 CACNA1D
Dre 8 cacna1db
CACNA1D
Tni 9 1Db
Ola 7 1Db
Gac XII 1Db
Dre 11 cacna1da
Tni 11 1Da
Ola 5 1Da
Gac s27 1Da
Aca 2 1F
Mdo X 1F
Mmu X Cacna1f
Hsa X CACNA1F
Dre 8 cacna1f
CACNA1F
Gac 1Fa
Ola 7 1Fa
Tni Un 1Fa
Dre 8 1Fb
Tni 9 1Fb
Gac XII 1Fb
Ola 7 1Fb
0.4

FIGURE S8
