# Supplementary material for: The vertebrate ancestral repertoire of visual opsins, transducin alpha subunits and oxytocin/vasopressin receptors was established by duplication of their shared genomic region in the two rounds of early vertebrate genome duplications
Source: BMC Evol Biol. 2013 Nov 2;13:238. doi: 10.1186/1471-2148-13-238 (PMC3826523; doi:10.1186/1471-2148-13-238)
Supplement: Additional file 4 — Supplementary notes. Topologies of neighboring gene family trees. [file 1471-2148-13-238-S4.pdf]

SUPPLEMENTARY NOTES

Topologies of neighboring gene family trees

Our conserved synteny analysis is based on the phylogenetic analyses of 34 neighboring gene families (table 2). The phylogenetic analyses of most these gene families were rooted with identified fruit fly family members. Where no fruit fly sequence could be identified, the trees were rooted with *C. elegans* family members (CDK, SYP and TIMM17), the identified tunicate family members (KLHDC, LRRN, PHTF, PLG and SRGAP), or midpoint-rooted in the lack of a putative invertebrate family member (B4GALNT, PTPN, RBM and SEMA3).

Out of the 34 neighboring gene families, 23 support duplications in the time-window of the 2R events. These gene families are ATP2B1, CAMK1, CELSR, CNTN, COPG, FLN, IKBKE, IQSEC, KDM, KLHDC, L1CAM, LRRN, MAGI, PLG, PLXNA, PPM, PRICKLE, RSBN, SRGAP, TIMM17, TWF, UBA and USP. These families show an early vertebrate divergence of two to four well supported clusters including both tetrapod and teleost sequences in most cases, with one single tunicate or lancelet family member diverging basal to the vertebrate subtype clusters. In the KDM family NJ tree the identified tunicate sequence branches basal to only two out of three subtype clusters. However, this topology has low bootstrap support. The LRRN, CNTN and MAGI families have additional teleost clusters with unresolved or unknown orthology relationships to tetrapod sequences, while the PPM family has one cluster that lacks teleost sequences. The USP family is presented with a midpoint root since there are two identified tunicate family members that separate the tree into two main vertebrate branches. The larger of these two branches has two clusters including both tetrapod and teleost sequences diverging in the time-window of the 2R events. The CNTN family is a special case since there are three clusters (CNTN3, CNTN4 and CNTN6) that seem to represent ancient local duplications shared by both tetrapods and teleosts. The orthology relationship between tetrapods and teleost for these ancient local duplicates is better resolved in the PhyML tree than in the NJ tree, however there is one cluster of teleosts sequences with unclear orthology in both NJ and PhyML trees.

With regard to the teleosts-specific tetraploidization (3R), the ATP2B, CAMK, CNTN, FLN, IQSEC, KDM, L1CAM, MAGI, PLXNA, PPM, PRICKLE, RSBN and TWF families have duplicate teleost branches that diverge in the time-window of the 3R event within at least one of the vertebrate clusters. These duplicate teleost branches have well-supported and clear topologies in both the NJ and PhyML trees for the ATP2B1, FLN, KDM, PLXNA, RSBN and TWF families, or in at least one of them for the CAMK, CNTN, IQSEC, L1CAM, MAGI, PPM, PRICKLE and SRGAP families. The most common departure from a clear 3R-generated duplicate topology is the divergent clustering of individual zebrafish sequences as outliers. The LRRN family also seems to have duplicate teleost branches, however the divergences of these are unclear in both the NJ tree and the PhyML tree.

In the tables showing the conserved synteny between the human, chicken, zebrafish and stickleback chromosome blocks (Supplementary data 4), dashed cells indicate divergences between the NJ tree and PhyML tree topologies and white cells indicate teleost sequences with unclear orthology relationships in the phylogenetic analyses. Alignments and phylogenetic trees for the neighboring gene families are provided as a citable file set with a stable identifier, available from: http://dx.doi.org/10.6084/m9.figshare.705852.

Aside from the 23 neighboring gene families described above, four gene

families are consistent with the 2R events, but have no invertebrate family members. Therefore the time-window of their divergence is unknown. The B4GALNT, PTPN and RBM families have tree topologies with two or three well-supported clusters including both tetrapod and teleost sequences. The SEMA3 family is a special case since there seem to have been ancestral local duplications before the 2R events, giving rise to seven subtype clusters with both tetrapod and teleost sequences. The relationships between the subtype clusters is not clear comparing the NJ tree and the PhyML tree, even though all subtype clusters are well-supported. There are also additional teleost-specific clusters with unclear orthology relationships.

The B4GALNT and RBM families have clusters with duplicate teleost branches that are consistent with duplications in the 3R event in both the NJ and PhyML trees. For the SEMA3 family, some subtype clusters have duplicate teleost branches that support duplication in 3R, while some are unclear.

The remaining seven neighboring gene families have clusters that are unresolved with regard to the divergence between tetrapods and teleosts (CDK, ERC, PHTF, SYP, WNK) and/or unresolved branching of the identified tunicate or lancelet sequences (CACNA2D, CDK, ERC, GXYLT). Often the topologies are inconsistent between the NJ trees and the PhyML trees. The CDK, PHTF and SYP families also have clusters of teleost sequences with unclear orthology relationships. The locations of the gene members of these families were nonetheless noted in order to investigate whether they are consistent with the involvement of the vertebrate tetraploidizations.

Some of the inconclusive families are worth describing in closer detail. The CACNA2D PhyML tree is consistent with two CACNA2D sub-families expanding in the time-window of 2R, each with a corresponding tunicate sequence clustering basal to two vertebrate clusters. However, in the NJ tree both tunicate sequences branch basal to one of the sub-families, making the time-window of the divergence unclear. For three of the CACNA2D subtype clusters, the PhyML tree is consistent with duplications in 3R. This is unclear in the NJ tree. The WNK family NJ and PhyML trees, while lacking a tunicate or lancelet branch, are consistent with the early divergence of four clusters including both tetrapod and teleost sequences when taken together. One cluster has poor statistical support in the PhyML tree, and two other clusters are not resolved with regard to species taxonomy in the NJ tree.

Several of the inconclusive gene families also have individual clusters that have duplicate teleost branches consistent with duplications in the 3R event. This includes the PhyML trees of the aforementioned CACNA2D and WNK families, but also the CDK, ERC and SYP families.
